# Dissecting wheat epitranscriptome and proteome under salt stress characterizes an m^6^A reader gene vital for salinity adaptation

**DOI:** 10.1093/plphys/kiag540

**Published:** 2026-07-24

**Authors:** Jie Zang, Qian Zhang, Yuyu Zhang, Zheng Wang, Yuxiu Dong, Yongming Chen, Xian Sheng Zhang, Yifeng Hou

**Affiliations:** State Key Laboratory of Wheat Improvement, Peking University Institute of Advanced Agricultural Sciences, Shandong Laboratory of Advanced Agricultural Sciences in Weifang, 699 Binhu Road, Weifang, Shandong 261325, China; State Key Laboratory of Wheat Improvement, Peking University Institute of Advanced Agricultural Sciences, Shandong Laboratory of Advanced Agricultural Sciences in Weifang, 699 Binhu Road, Weifang, Shandong 261325, China; State Key Laboratory of Wheat Improvement, Peking University Institute of Advanced Agricultural Sciences, Shandong Laboratory of Advanced Agricultural Sciences in Weifang, 699 Binhu Road, Weifang, Shandong 261325, China; State Key Laboratory of Wheat Improvement, Peking University Institute of Advanced Agricultural Sciences, Shandong Laboratory of Advanced Agricultural Sciences in Weifang, 699 Binhu Road, Weifang, Shandong 261325, China; State Key Laboratory of Wheat Improvement, College of Life Sciences, Shandong Agricultural University, 61 Daizong Road, Tai'an, Shandong 271018, China; State Key Laboratory of Wheat Improvement, College of Life Sciences, Shandong Agricultural University, 61 Daizong Road, Tai'an, Shandong 271018, China; State Key Laboratory of Wheat Improvement, Peking University Institute of Advanced Agricultural Sciences, Shandong Laboratory of Advanced Agricultural Sciences in Weifang, 699 Binhu Road, Weifang, Shandong 261325, China; State Key Laboratory of Wheat Improvement, College of Life Sciences, Shandong Agricultural University, 61 Daizong Road, Tai'an, Shandong 271018, China; State Key Laboratory of Wheat Improvement, Peking University Institute of Advanced Agricultural Sciences, Shandong Laboratory of Advanced Agricultural Sciences in Weifang, 699 Binhu Road, Weifang, Shandong 261325, China

## Abstract

Soil salinization is a major abiotic stress limiting wheat production. Although transcriptional responses to salt stress are well-studied, the role of posttranscriptional regulation, particularly through RNA modifications, remains unclear in wheat (*Triticum aestivum* L.). Here, we present an integrated analysis of the early salt stress response using Nanopore direct RNA sequencing and quantitative proteomics. We generated genome-wide maps of *N*^6^-methyladenosine (m^6^A) modifications, concurrently profiling alternative polyadenylation events and poly(A) tail length dynamics. This multiomics approach characterizes coordinated epitranscriptomic reprogramming and enabled the construction of a regulatory network linking m^6^A marks to proteomic changes. Furthermore, we identified and functionally validated the putative m^6^A reader protein EVOLUTIONARILY CONSERVED C-TERMINAL REGION 5 (TaECT5) as a positive regulator of wheat salt tolerance. Our study provides a systems-level view of posttranscriptional regulation during salt stress in wheat and identifies potential targets for enhancing salt tolerance.

## Introduction

Soil salinization stands as a major abiotic stress that constrains global agricultural productivity and poses a significant threat to food security. As a staple food crop, wheat is susceptible to salt stress, which substantially reduces its yield and limits suitable cultivation areas (van Zelm et al. 2020; Mao et al. 2023; Liang et al. 2024). This stress primarily inflicts damage through ionic toxicity, osmotic stress, and secondary oxidative stress. To mitigate these effects, wheat has evolved a multilayered array of adaptive mechanisms (Mao et al. 2023; Liang et al. 2024). These physiological adaptations primarily involve regulating ion homeostasis by extruding excess Na^+^ from cells via the Na^+^/H^+^ antiporter Salt Overly Sensitive 1 (TaSOS1) and removing Na^+^ from the xylem stream through High-affinity K^+^ Transporter 1 (HKT1)-type proteins such as TaHKT1;5-D (Byrt et al. 2014; Zheng et al. 2022; Wang et al. 2024). Cellular homeostasis is further maintained through osmotic adjustment via the accumulation of compatible solutes, coupled with the activation of antioxidant enzyme systems to scavenge harmful reactive oxygen species (ROS) (Mao et al. 2023; Liang et al. 2024). Such adaptive responses are underpinned by complex intracellular signaling cascades triggered by salt stress. The resultant transcriptional reprogramming is coordinated by key transcription factor families, including the basic leucine zipper (bZIP), NAC (named from NAM, ATAF, and CUC), and plant AT-rich sequence- and zinc-binding protein (PLATZ) families, which orchestrate the expression of tolerance-related genes (Huang et al. 2015; Sun et al. 2015; Xu et al. 2015; Bi et al. 2021; Zhang et al. 2023; Wei et al. 2025). Beyond transcriptional control, posttranscriptional regulatory mechanisms are now known to play crucial roles (Singh and Roychoudhury 2021; Huang et al. 2024). However, the posttranscriptional regulatory network in wheat under salt stress, particularly governed by RNA modifications such as *N*^6^-methyladenosine (m^6^A), remains largely unexplored. Unraveling these mechanisms could open new avenues for enhancing wheat salt tolerance.

RNA modifications constitute a crucial layer of gene expression regulation (Sharma et al. 2023; Shen et al. 2023). m^6^A is the most prevalent, dynamic, and reversible internal modification on eukaryotic messenger RNA (mRNA), governing nearly its entire lifecycle (Tang et al. 2023; Cappannini et al. 2024; Nguyen and Kang 2024). This intricate regulation is executed by a dedicated suite of effector proteins: “writers” (methyltransferases) install the mark, “erasers” (demethylases) remove it, and “readers” (recognition proteins) interpret it to direct downstream molecular outcomes (Yue et al. 2019; Shen et al. 2023; Tang et al. 2023; Nguyen and Kang 2024; Song et al. 2024). In plants, m^6^A plays a well-established role in developmental programming and adaptation to environmental stress such as salinity (Hu et al. 2022; Tang et al. 2023; Song et al. 2024; Cai et al. 2025). Genome-wide studies across species have revealed that salt stress triggers profound and extensive reprogramming of the m^6^A epitranscriptome, underscoring its functional importance (Hu et al. 2021; Zheng et al. 2021; Wang et al. 2022a, 2022b; Wang et al. 2022a, 2022b; Li et al. 2023; Liu et al. 2024a, 2024b, 2024c; Zhao et al. 2024; Qian et al. 2025; Zhang et al. 2025). This remodeling is precisely controlled by the balanced activity of writers and erasers (Huong et al. 2020; Hu et al. 2021; Shoaib et al. 2021; Amara et al. 2022; Cai et al. 2024a, 2024b; Zhao et al. 2024; Zheng et al. 2024; Wang et al. 2025a, 2025b, 2025c, 2025d; Wang et al. 2025a, 2025b, 2025c, 2025d; Zhang et al. 2025). In *Arabidopsis*, for instance, salt stress upregulates core writer components such as VIRILIZER (VIR) and FIONA1 (FIO1), which catalyzed m^6^A deposition that is critical for salt tolerance by modulating the mRNA stability of key stress-responsive transcripts (Hu et al. 2021; Cai et al. 2024a, 2024b). Interestingly, eraser proteins within the AlkB homolog family exert varying effects on salt tolerance, as exemplified by the decreased tolerance in the *atalkbh9c* mutant versus the enhanced tolerance in *atalkbh6* and *atalkbh10b* mutants (Huong et al. 2020; Shoaib et al. 2021; Amara et al. 2022). This functional divergence is likely attributable to their removal of m^6^A from distinct sets of target mRNAs. At the decoding level, reader proteins also fulfill crucial roles (Wang et al. 2022a, 2022b; Amara et al. 2024; Cai et al. 2024a, 2024b; Nguyen et al. 2025). In *Arabidopsis*, the reader protein EVOLUTIONARILY CONSERVED C-TERMINAL REGION 8 (ECT8), which contains an m^6^A-binding YT521-B homology (YTH) domain, enhances salt tolerance by degrading mRNAs that encode negative regulators while stabilizing positive regulator transcripts (Cai et al. 2024a, 2024b; Nguyen et al. 2025). Its homolog ECT12 also contributes to salt adaptation by stabilizing transcripts involved in the stress response (Amara et al. 2024). In crops such as cotton, the expression of the putative reader *GhECT6* is induced by salt stress and is essential for tolerance (Wang et al. 2022a, 2022b). However, how this regulatory network operates under salt stress in wheat remains to be elucidated.

Alternative polyadenylation (APA) is a pivotal posttranscriptional regulatory mechanism for plant adaptation to salt stress (Tian and Manley 2017; Téllez-Robledo et al. 2019; Ma et al. 2022; Wang et al. 2025a, 2025b, 2025c, 2025d). Transcriptome-wide studies indicate that m^6^A in plant mRNAs is predominantly enriched in the 3′ untranslated region (3′ UTR) and around the stop codon regions also critical for APA regulation—suggesting a functional convergence between m^6^A modification and APA (Luo et al. 2014; Tian and Manley 2017). Subsequent mechanistic studies, such as those involving the m^6^A writer component VIR and the reader protein CPSF30-L, have established m^6^A as a key regulator of APA (Parker et al. 2020; Hou et al. 2021; Hu et al. 2021; Song et al. 2021). For example, loss of VIR function significantly reduces m^6^A levels specifically in the 3′ UTR. This reduction shifts genome-wide poly(A) site selection toward proximal sites, providing direct evidence for the central role of m^6^A in APA (Parker et al. 2020). Furthermore, by mediating 3′ UTR m^6^A deposition to modulate APA, VIR fine-tunes the mRNA stability of key negative regulators of salt stress, a mechanism essential for plant salt tolerance (Hu et al. 2021). Additionally, CPSF30-L, which integrates m^6^A recognition with polyadenylation activity, participates in m^6^A-dependent APA site selection in response to signals such as nitrate and ABA (Hou et al. 2021; Song et al. 2021). These findings imply that m^6^A cooperates with the APA machinery to control mRNA 3′-end fate under salt stress. Beyond APA, poly(A) tail length (PAL) dynamics represent another regulatory layer with emerging links to m^6^A (Li et al. 2022; Passmore and Coller 2022; Song et al. 2023; Qian et al. 2025). Nevertheless, how salt stress coordinately regulates APA events with m^6^A modification, or how PAL dynamics are associated with m^6^A, remains unclear in wheat.

Building on the established functional convergence and mechanistic links among m^6^A, APA, and PAL, a systems-level understanding of m^6^A function requires not only mapping its genomic distribution but, more crucially, elucidating its ultimate impact on protein expression (Fan and Shen 2025). To that end, Nanopore direct RNA sequencing (DRS) enables the simultaneous detection of RNA modifications (including m^6^A), identification of APA sites, and measurement of PAL, thereby providing a comprehensive methodology for profiling the dynamic epitranscriptome (Zhu et al. 2024). Importantly, strong evidence positions m^6^A as a key regulator of the proteome (Gao et al. 2022; Li et al. 2024; Yang et al. 2025). For example, in rice, m^6^A levels in 3′ UTRs negatively correlate with protein abundance and drive proteomic differences (Li et al. 2024). Given this established regulatory role, integrating detailed m^6^A maps from DRS with quantitative proteomic data is essential for constructing the complete RNA-to-protein regulatory network and revealing the mechanisms of plant stress adaptation. However, despite this rationale, the potential correlation among these dynamics in wheat under salt stress has not been explored through an integrated DRS–proteomics framework.

In this study, we integrated Nanopore DRS with quantitative proteomics to systematically profile posttranscriptional regulatory dynamics during early salt stress in wheat. Our analysis included genome-wide mapping of m^6^A modifications, concurrent assessment of APA and PAL variations, and the construction of an association network linking the epitranscriptome and proteome. Furthermore, we identified and functionally validated a key salt stress-induced putative m^6^A reader gene, *TaECT5*. Together, this work delineates how epitranscriptomic mechanisms coordinate the early salt stress response in wheat and identifies potential targets for future precision breeding strategies.

## Results

### Rapid transcriptional and physiological responses define the early salt stress stage in wheat roots

Plants respond to salt stress through a phased process encompassing perception, early signaling, downstream signaling, and adaptive responses (van Zelm et al. 2020). To dissect the epitranscriptomic regulation underlying the earliest phase in wheat, we first aimed to define a time point that captures the associated rapid molecular changes. We therefore monitored the expression of 15 well-characterized salt response related genes in seedling roots using reverse transcription quantitative polymerase chain reaction (RT-qPCR) at 0, 0.5, 1, 3, 6, 12, and 24 h post-salt treatment (hpt). Notably, 11 of 15 genes exhibited significantly differential expression as early as 0.5 hpt (Figure S1), indicating rapid transcriptional reprogramming. This early transcriptional shift suggested that the 0.5 hpt time point could serve as a promising candidate for capturing the initiating regulatory phase.

**Figure 1:**
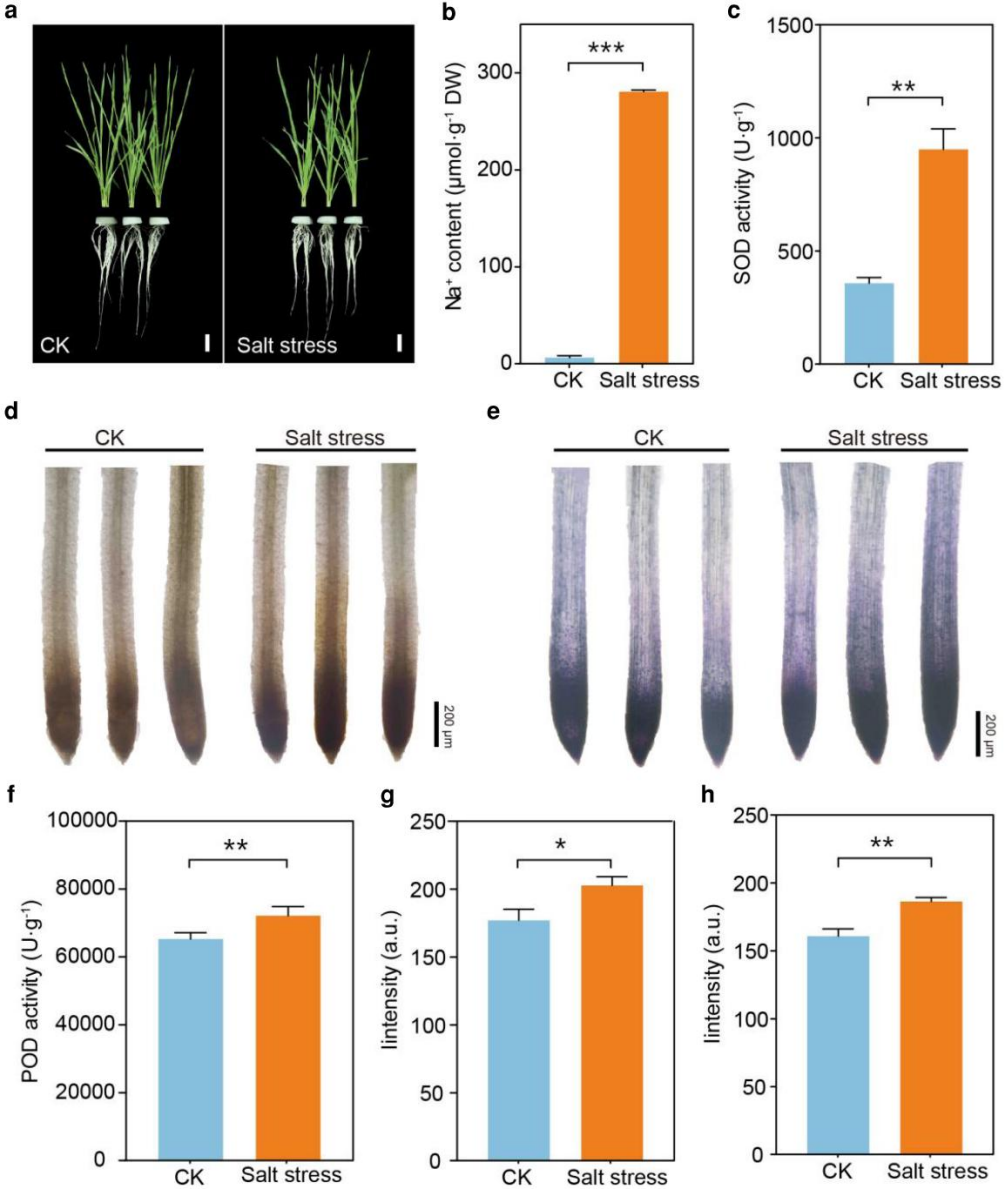
Phenotypic analysis of wheat seedlings under normal and salt stress conditions. (a) Observation of wheat seedlings under CK and salt stress conditions. (b) Analysis of Na^+^ accumulation in CK and salt stress seedling roots. (c) Measurement of SOD activity in CK and salt stress seedling roots. (d and e) DAB and NBT staining in CK and salt stress seedling roots, respectively. (f) Measurement of POD activity in CK and salt stress seedlings roots. (g–h) Quantified results of (d) and (E), respectively. Scale bar, 1 cm in (A); 200 μm in (d) and (e). Data in (b), (c), and (f) are means ± standard deviation (SD) from 3 replicates, with 10 individual plants per replicate. Asterisks indicate significant differences by Student's *t*-test (* *P* < 0.05, ** *P* < 0.01, *** *P* < 0.001). a.u.: arbitrary units.

Salt stress triggers a series of early events, including ion homeostasis disruption and oxidative burst (van Zelm et al. 2020). To evaluate whether the transcriptional response at the candidate time point corresponds to these physiological hallmarks, we measured and compared key physiological parameters between control (CK, 0 hpt) and salt-stressed (salt stress, 0.5 hpt) roots. No visible phenotypic difference was observed between CK and salt stress seedlings (Fig. 1a). Quantification revealed a significant accumulation of Na^+^ in roots exposed to salt stress for 0.5 h relative to CK (Fig. 1b). Consistently, nitroblue tetrazolium (NBT) and 3,3′-diaminobenzidine (DAB) staining assays detected robust signals corresponding to O^2^- and H_2_O_2_ accumulation, respectively, in salt-stressed seedling roots (Fig. 1d and e, g and h). Furthermore, enzymatic activity assays demonstrated that the activities of peroxidase (POD) and superoxide dismutase (SOD), 2 key antioxidant enzymes, were significantly higher in salt-stressed roots compared to CK (Fig. 1c and f). Collectively, these physiological and biochemical data confirm that 0.5 h of salt stress is sufficient to induce the defining early responses, thereby validating this time point as suitable for subsequent epitranscriptomic analysis of the initial regulatory mechanisms.

### High-quality Nanopore direct RNA sequencing (DRS) data from salt-stressed wheat roots

**Figure 2:**
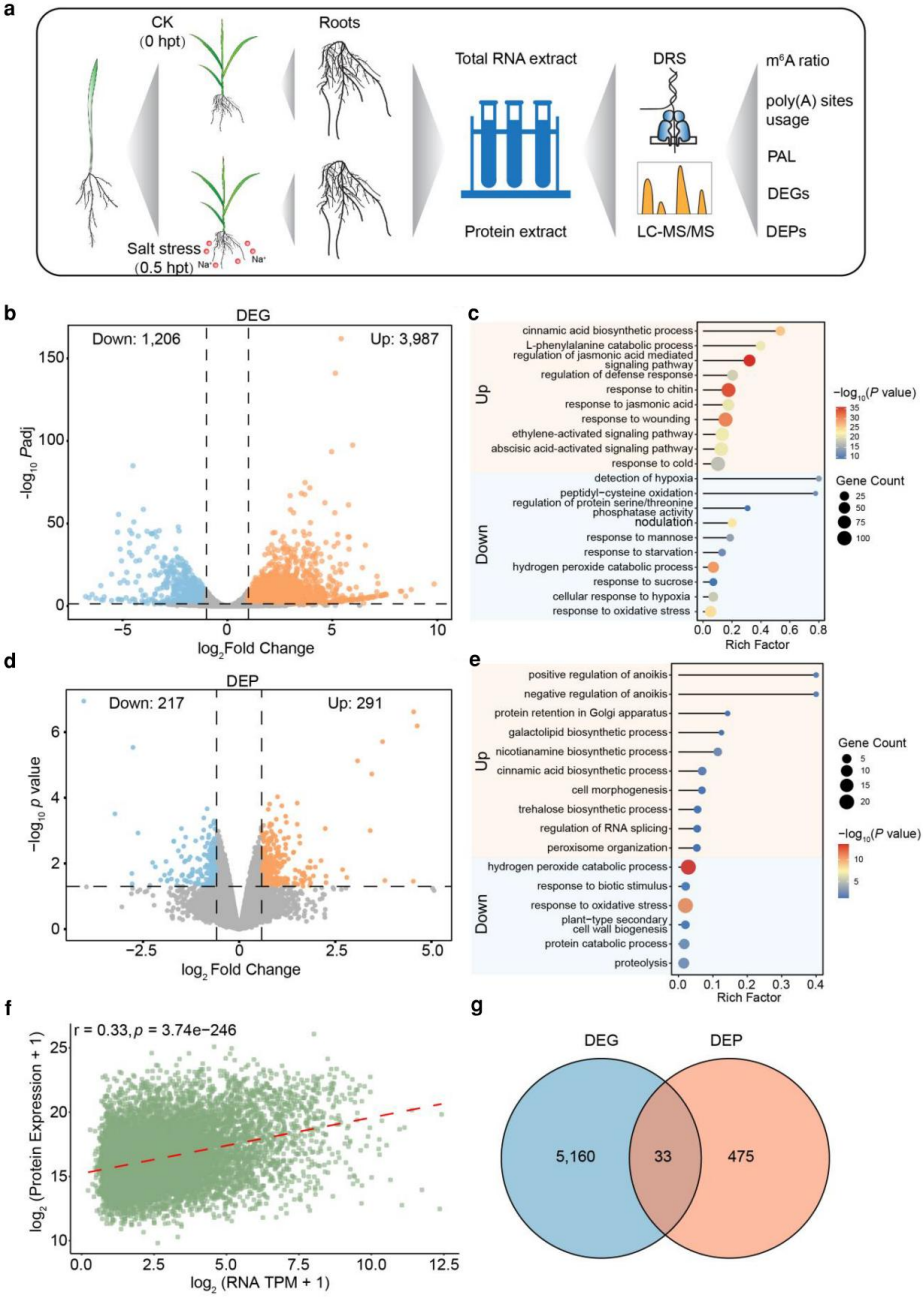
DEPs showed overlap with DEGs upon salt stress. (a) Flowchart for analysis of epitranscriptome and proteome changes triggered by salt stress using Nanopore DRS and 4D-DIA. (b) Volcano plots showing DEGs under salt stress. (c) GO enrichment analysis of DEGs under salt stress. (d) Volcano plots showing DEPs under salt stress. (e) GO enrichment analysis of DEPs under salt stress. (f) The correlation between gene expression and protein abundance. (g) Venn diagram shows the overlap between DEGs and DEPs.

Building upon the identified early salt stress phase, we sought to characterize the associated epitranscriptomic changes. To profile the epitranscriptomic landscape of wheat seedling roots in response to salt stress, DRS was performed on root samples collected under CK and salt-stressed conditions (Fig. 2a) (see materials and methods). Per-sample sequencing output reached ∼10 to 11 billion bases, with each library generating 11.6 to 17.6 million reads and an average read length of 600 to 910 nt. Following base calling and quality control, ∼89.32% of the DRS reads were successfully aligned to the wheat reference genome (IWGSC Refseq v1.1). Of these, about 90% mapped confidently to annotated transcripts in the reference transcriptome, indicating good overall data quality and alignment efficiency for downstream epitranscriptomic analysis.

### Distinct and coordinated transcriptomic and proteomic responses to salt stress in wheat roots

To profile the transcriptional response during the early salt stress phase, we identified differentially expressed genes (DEGs) from the DRS data. In total, 5,193 DEGs were detected across the wheat transcriptome in response to salt stress, comprising 3,987 upregulated and 1,206 downregulated genes (Fig. 2b). Functional enrichment analysis revealed that upregulated DEGs were significantly enriched in plant hormone signaling pathways, such as jasmonic acid- and abscisic acid-activated signaling pathway (Fig. 2c), highlighting the rapid activation of hormonal signaling. In contrast, downregulated DEGs were enriched in oxidation reduction processes, including responses to oxidative stress and hydrogen peroxide catabolism (Fig. 2c). This is consistent with the elevated ROS levels observed in salt stress seedling roots. We further focused on DEGs annotated to “response to salt stress” term, identifying 123 such genes. Notably, 87.8% of them were upregulated, encompassing key transcription factor families: NAC, WRKY, ethylene-responsive transcription factor (ERF), bZIP, and ABA-responsive binding factor (ABF). Consistent with previous reports in cereals, NAC and bZIP family members were particularly strongly induced (Liu et al. 2024a, 2024b, 2024c; Zhang et al. 2025).

mRNA and protein abundance exhibit widely varying associations across distinct organisms, cell types, and functional categories (Greenbaum et al. 2003; de Sousa Abreu et al. 2009; Ponnala et al. 2014). Given this well-documented but context-dependent discordance, we performed parallel quantitative proteomic analysis to identify differentially expressed proteins (DEPs) under salt stress (Fig. 2a). A total of 508 DEPs were identified, with 291 upregulated and 217 downregulated (Fig. 2d). Gene Ontology (GO) enrichment analysis indicated that upregulated proteins were mainly enriched in carbohydrate metabolic process and amino acid metabolic process (Fig. 2e). Downregulated proteins were primarily enriched in oxidation reduction processes, consistent with the GO analysis result at transcriptional level (Fig. 2c and e). Peroxidases are a core component of the cellular ROS scavenging system and play pivotal roles in mediating plant salt tolerance (Jin et al. 2019). Notably, all 21 downregulated proteins annotated to the “response to oxidative stress” were identified as POD (Fig. 2e). This observation suggested that the coordinated downregulation of POD proteins may contribute to a transient ROS burst, which in turn acts as an amplified signaling hub to potentiate the activation of downstream salt stress-responsive pathways. To integrate the 2 layers of regulation, we examined the correlation between changes in transcript and protein abundance. Overall, a significant but weak positive correlation was observed (r = 0.33, *P* = 3.74e-246) (Fig. 2f), underscoring the importance of posttranscriptional regulation. Further analysis identified a subset of 33 genes with concordant differential expression at both the RNA and protein levels (Fig. 2g), indicating points of convergent regulatory control during the early salt stress response.

### Dynamic changes of m^6^a modification under salt stress

**Figure 3:**
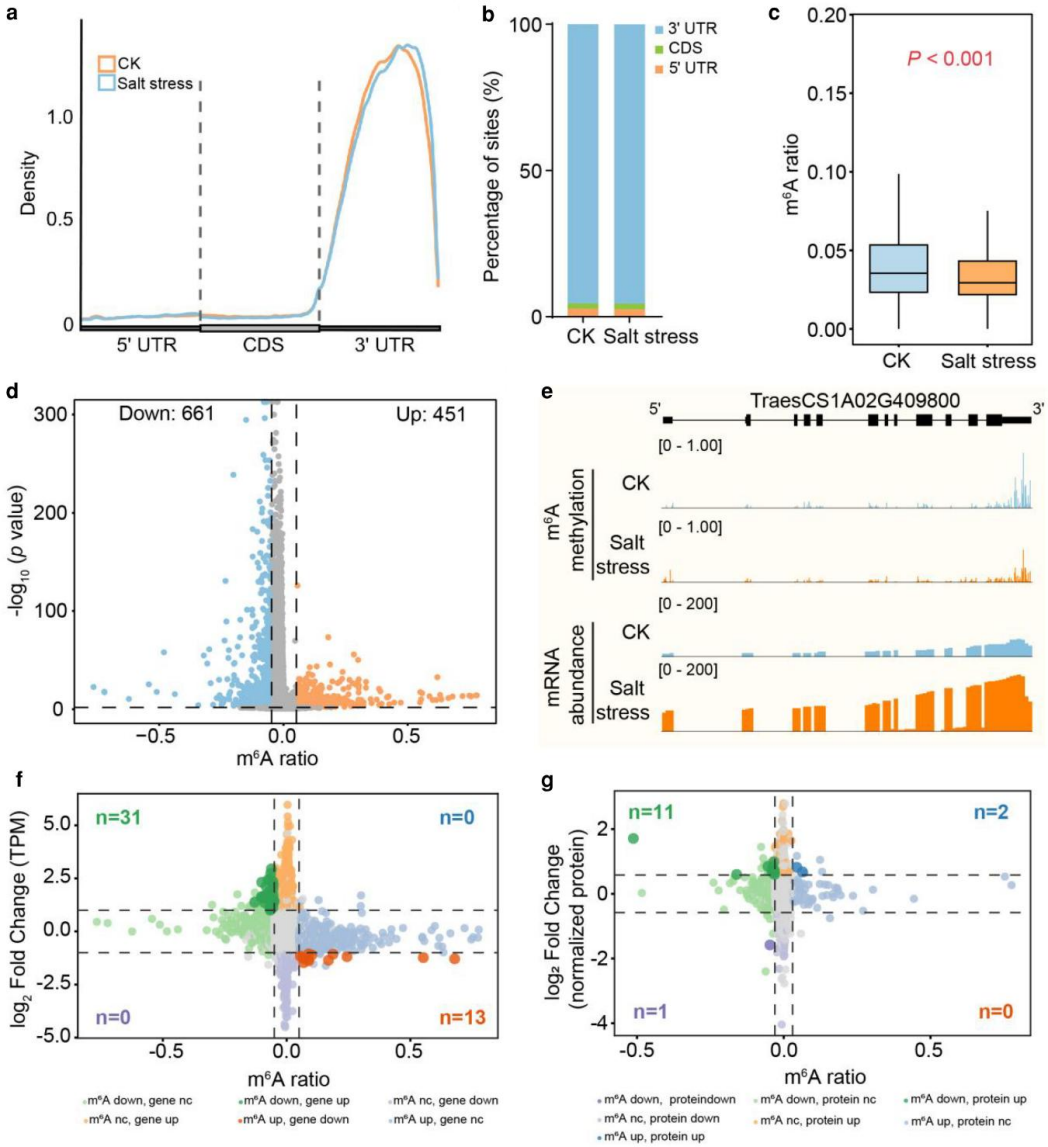
Salt stress-induced dynamic changes of m^6^A modification. (a) Metagene profile shows the distribution of m^6^A sites across coding genes under normal and salt stress conditions. (b) Histogram shows the percentage of m^6^A sites in the annotated gene region including 3′ UTR, 5′ UTR, and CDS under normal and salt stress conditions. (c) Boxplot shows m^6^A ratio under normal and salt stress conditions. Boxes denote the interquartile range, where the bottom and top boundaries correspond to the 25th and 75th percentiles, respectively. The horizontal line within each box represents the median value, and whiskers extend to the minimum and maximum values of the dataset. Statistical significance was assessed using the Student's *t*-test. (d) Volcano plots show m^6^A DMSs in response to salt stress. (e) Differential m^6^A methylation patterns and mRNA expression profiles of the transcription factor *TaGBF1*. (f and g) Nine-quadrant scatter plots show associations of m^6^A methylation changes with gene expression and protein abundance, respectively.

To investigate whether the early salt stress response involves epitranscriptomic reprogramming, we detected m^6^A sites at single-base resolution using DRS data. A total of 159,705 and 130,573 m^6^A sites among 31,870 and 28,642 genes were identified in CK and salt stress samples, respectively (Figure S2a). Characterizing the m^6^A profiles revealed that m^6^A peaks were mainly located in 3′ UTR, accounting for over 95% of all identified peaks across all samples (Fig. 3a and b). Motif analysis of m^6^A modification sites was performed via the MEME software package, revealing RRACH (R = A/G, H = A/U/C) as the consensus sequence in CK and salt stress samples (Figure S2b).

Under salt stress, the average m^6^A modification ratio was significantly reduced in wheat roots (Fig. 3c). To dissect the salt-responsive m^6^A regulatory landscape, we identified m^6^A differentially methylated sites (m^6^A DMSs) through comparative profiling of CK and salt stress samples. In total, 451 genes showed an increased methylation and 661 genes showed a decreased methylation (Fig. 3d). Gene Ontology enrichment analysis showed that genes with elevated m^6^A levels were primarily enriched in terms related to DNA metabolic processes, organelle organization, and cell cycle progression, while genes with reduced m^6^A levels were mainly enriched in zeaxanthin epoxidase activity and ATP binding (Figure S2d). To further dissect the cross talk between salt stress and m^6^A modification, we collected well-characterized salt-responsive genes in wheat and systematically profiled their m^6^A modification dynamics. Intriguingly, our data demonstrated a prevalent reduction in m^6^A levels of these salt-responsive genes under early-stage salt stress (Figure S2c), indicating that dynamic m^6^A modification acts as a rapid epigenetic switch to orchestrate early salt stress adaptation in wheat. For instance, m^6^A methylation of *TaGBF1* (TraesCS1A02G409800), a well-documented negative regulator of salt tolerance in wheat (Sun et al. 2015), was significantly diminished under salt stress conditions (Fig. 3e).

To explore the interplay between m^6^A modification and gene expression dynamics, we integrated our m^6^A DMS data with transcriptomic profiling, identifying 44 DEGs that harbor m^6^A DMSs. These genes were stratified into 4 distinct regulatory patterns: 31 genes with elevated m^6^A modification levels showed a concurrent downregulation in expression, while another 13 genes with reduced m^6^A methylation were accompanied by upregulated expression (Fig. 3f). In the “m^6^A hypomethylation-coupled transcriptional upregulation” regulatory pattern, *TaGBF1* was recurrently identified (Fig. 3e). A suite of salt stress-responsive genes, including those encoding zeaxanthin epoxidase (a key enzyme in ABA biosynthesis; TraesCS3B02G141400 and TraesCS2B02G335400) and leucine-rich repeat receptor-like protein kinases (LRR-RLKs, critical for stress signal perception; TraesCS7D02G129800), showed the same trend as *TaGBF1*. Notably, no DEGs were detected in the 2 reciprocal patterns: genes with elevated m^6^A methylation coupled with upregulated expression, or reduced m^6^A modification associated with downregulated expression, suggesting a predominant inverse correlation between m^6^A modification status and gene expression levels in the context of salt stress. Consistent with this observation, our analysis revealed a significant negative correlation between the m^6^A ratio and transcript abundance (*r* = −0.0826, *P* = 4.23e-247), which was further confirmed by cumulative fraction analysis (Figure S3a-c).

Extending this analysis to the proteomic level revealed a largely consistent translational output for the hypomethylated/upregulated transcripts (Figure S3d-f). Among the 13 proteins with m^6^A hypomethylation, 12 exhibited concordant upregulation at the protein level, including functionally pivotal candidates directly linked to salt stress adaptation: mediator of RNA polymerase II transcription subunit 14 (TraesCS5A02G175600), callose synthase-like protein (TraesCS3A02G245500), and aminotransferase-related family protein (TraesCS7B02G246200). Only 1 protein (phosphoglucosamine mutase family protein, TraesCS5B02G105200) showed a discordant pattern, potentially reflecting additional posttranslational regulation or tissue-specific translational control (Fig. 3g). In contrast, the 2 proteins (Nck-associated protein and transmembrane protein, TraesCS7A02G268900 and TraesCS4A02G108500) originating from transcripts with hypermethylated sites were upregulated despite the transcriptional downregulation of their genes, suggesting that m^6^A hypermethylation may mediate context-dependent translational regulation that decouples protein abundance from mRNA levels (Figure 3g).

### Dynamic alterations in poly(A) site usage triggered by salt stress

**Figure 4:**
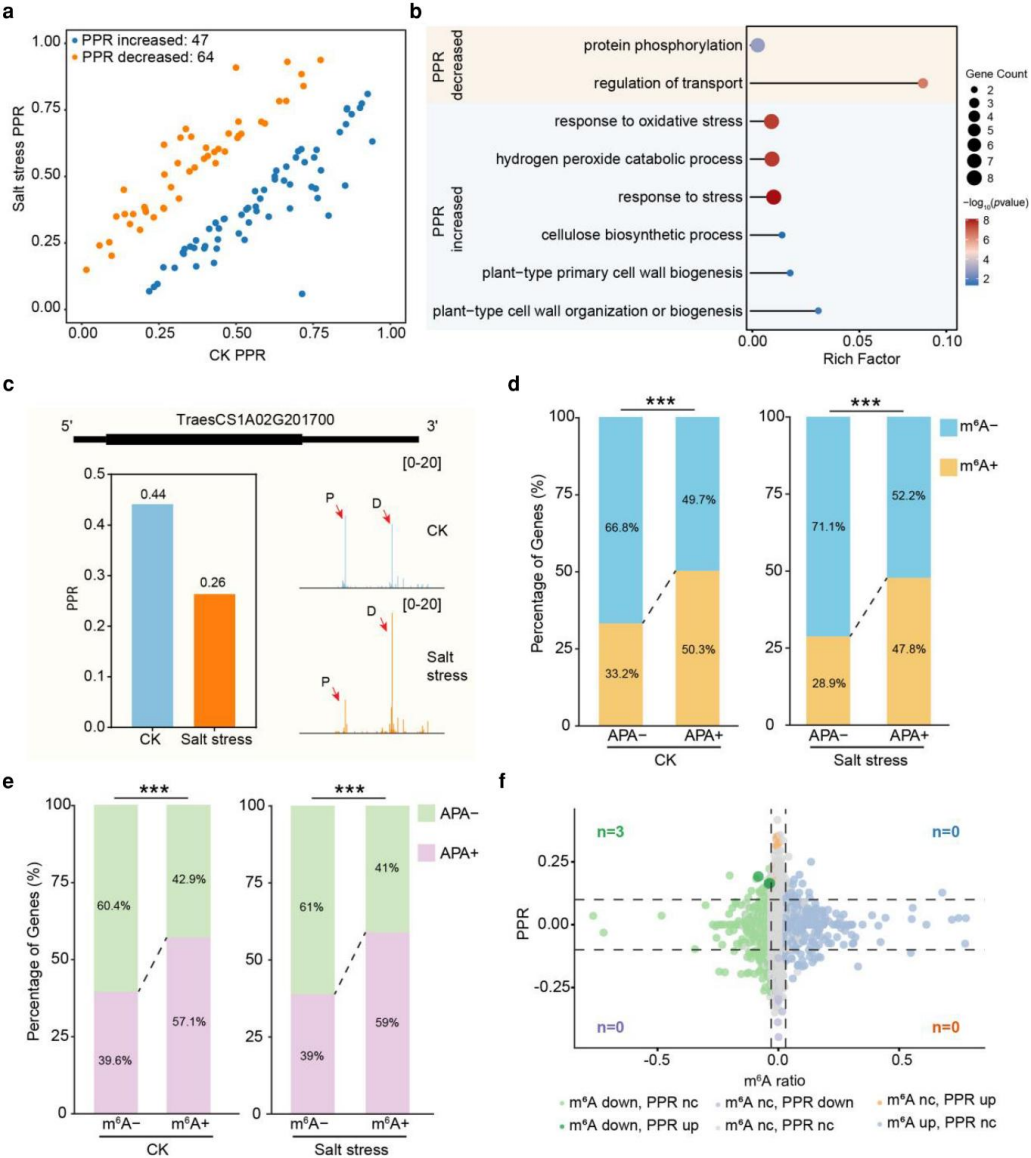
Poly(A) site usage dynamic changes under salt stress. (a) Scatter plots shows dynamic changes in PPR in response to salt stress. (b) GO enrichment analysis of differential PPR in response to salt stress. (c) Wiggle plot and histogram shows decreased PPR in *TaATL80*. (d) Proportion of m^6^A-modified transcripts with APA and non-APA events under normal and salt stress conditions. (e) Proportion of APA transcripts with m^6^A and non-m^6^A modification transcripts under normal and salt stress conditions. (f) Nine-quadrant scatter plot shows association of m^6^A methylation changes with differential APA. *P* values were calculated using the Fisher's exact test in (d) and (e), *** *P* < 0.001.

Polyadenylation is a critical and indispensable step in eukaryotic mRNA maturation, influencing stability, localization, and translation (Tian and Manley 2017). To characterize poly(A) site usage under salt stress, we compared the preference for proximal versus distal poly(A) sites by calculating the proximal poly(A) site usage ratio (proximal/distal + proximal, PPR) (Gao et al. 2022). In the salt stress vs. CK comparison, we observed 47 genes with increased PPR and 64 genes with decreased PPR (Fig. 4a), indicating a shift toward preferential usage of distal poly(A) sites under salt stress. A representative example is *TaATL80* (ARABIDOPSIS TOXICOS EN LEVADURA 80, TraesCS1A02G201700), a homolog of *ATL80* involved in stress response progress (Méndez-Gómez et al. 2024), which displayed a clear preference for the distal poly(A) site under salt stress conditions (Fig. 4c). Among the genes with differential APA events, 2 were annotated with the “response to salt stress” GO term. Functional enrichment analysis of salt-induced differential APA genes revealed that those with increased PPR were significantly enriched in response to stress and response to oxidative stress, such as *TaPRX52* (PEROXIDASE 52, TraesCS1A02G078100). In contrast, genes with decreased PPR were enriched in regulation of transport and protein phosphorylation, such as *TaATL80* and *TaRLK1* (RECEPTOR-LIKE KINASE 1, TraesCS4A02G267300) (Fig. 4b and c and Figure S4a and b).

Given the strong enrichment of m^6^A modification peaks in the 3′ UTR, we further investigated the potential correlation between m^6^A marks and APA events in wheat under salt stress. Our analysis showed that 50.3% of genes harboring at least 2 poly(A) sites were m^6^A-modified, which was significantly higher than the 33.2% m^6^A modification rate observed in genes without APA events. Conversely, 57.1% of m^6^A-modified genes were identified to carry APA events, a proportion substantially higher than the 39.6% observed in nonmodified genes. Importantly, this intimate association between m^6^A modification and APA site usage was consistently validated under salt stress condition (Fig. 4d and e). Collectively, these data reveal a strong correlation between m^6^A deposition and poly(A) site selection in wheat, both under normal and stress conditions. We also performed an integrated analysis of differential APA events and m^6^A modification. Among genes preferentially using proximal poly(A) sites, 3 genes exhibited significantly decreased m^6^A signals under salt stress (Fig. 4f). This coordinated change provides correlative evidence suggesting that m^6^A modification may be involved in regulating APA during the wheat salt stress response.

### Dynamic alterations in PAL triggered by salt stress

**Figure 5:**
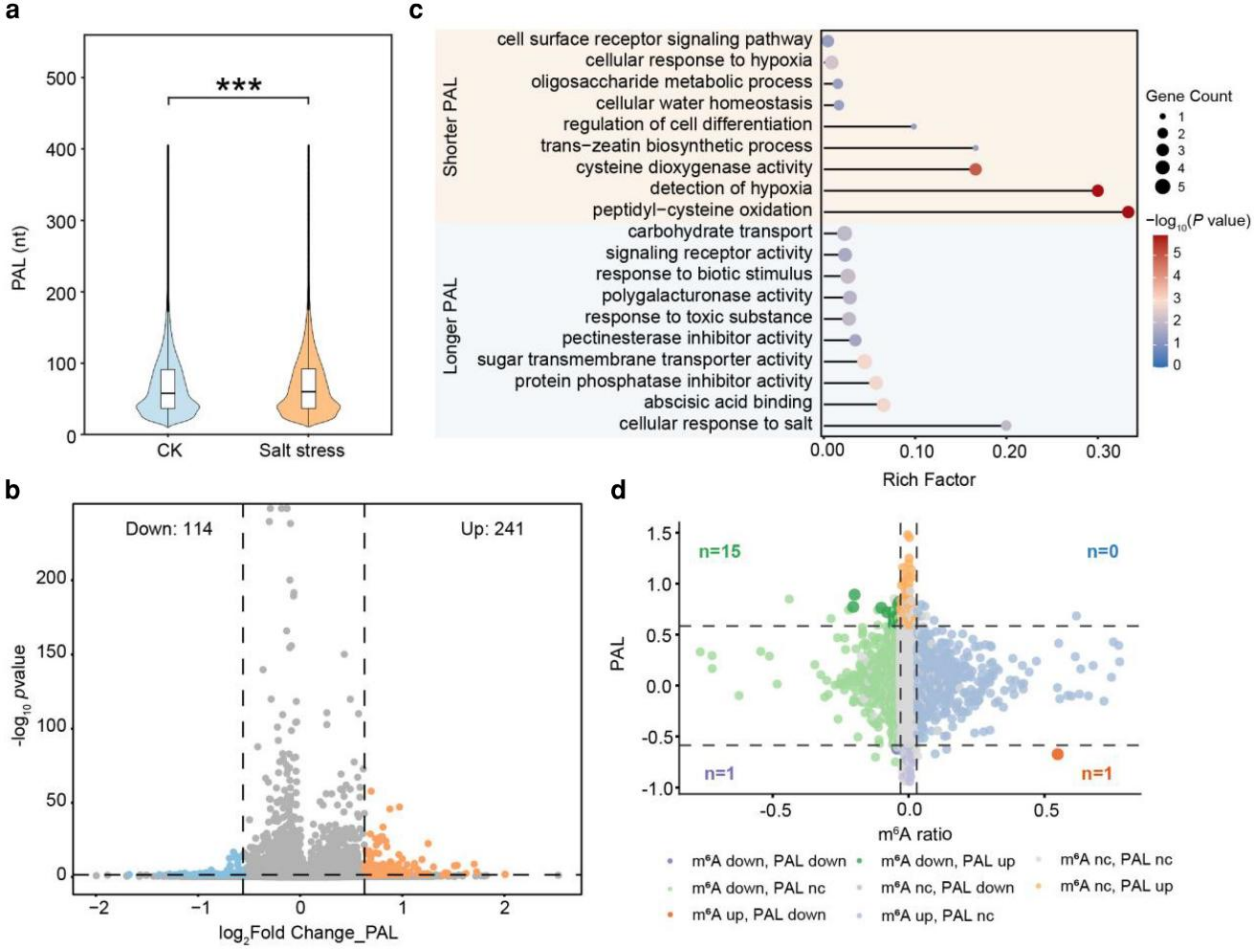
PAL dynamic changes under salt stress. (a) Average PAL of modified transcripts in CK and salt stress. Statistical significance was determined using a Mann–Whitney–Wilcoxon test. (b) Volcano plots show differential PAL in response to salt stress. (c) GO enrichment analysis of differential PAL under salt stress. (d) Nine-quadrant scatter plot shows association of m^6^A methylation changes with PAL.

Poly(A) tail length is a key determinant of mRNA stability and translational efficiency and is directly influenced by poly(A) site selection (Passmore and Coller 2022). Utilizing the full-length poly(A) tail sequences from DRS, we examined the dynamic changes in median PAL in response to salt stress. Consistent with a recent DRS-based study on salt stress (Qian et al. 2025), PAL was elongated under salt stress condition (Fig. 5a). We further identified 669 genes with salt stress-induced differential PAL changes, including 241 with elongated tails and 114 with shortened tails (Fig. 5b). Gene Ontology enrichment analysis showed that genes with longer PAL were enriched in cellular response to salt and abscisic acid binding, while genes with shorter PAL were enriched in cellular water homeostasis (Fig. 5c). For instance, the median PAL of TraesCS4B02G068100 (encoding a poly(A) polymerase) increased by 32.5 nt in salt stress relative to CK. A similar trend was observed in TraesCS1D02G265600 and TraesCS5B02G246600. Conversely, reduced PAL was detected in several genes, including TraesCS4A02G052600 and TraesCS5D02G078800, under salt stress conditions. Among all differential PAL events, we investigated genes related to the “response to salt stress” GO term and identified one gene showing longer PAL under salt stress condition.

Additionally, we also explore the relationship of PAL and gene expression abundance. As expected, highly expressed genes tended to have shorter PAL, whereas lowly expressed genes showed a preference for longer PAL (Figure S4c and d), in line with previous studies (Gao et al. 2022). Given the established link between m^6^A and gene expression in our study, we further investigated the potential association between PAL and m^6^A modification. The results revealed that m^6^A-modified genes exhibited longer PAL overall, and this trend was more pronounced under salt stress conditions (Fig. 5d, Figure S4e). Integration of differential m^6^A and differential PAL showed that m^6^A-modified genes were predominantly clustered in the region with longer PAL (eg m^6^A_down/PAL_up accounted for n = 15 genes). Collectively, these results reveal coordinated changes in PAL, gene expression, and m^6^A modification during the early salt stress response in wheat.

### TaECT5 contributes to the salt stress response pathway in wheat

**Figure 6:**
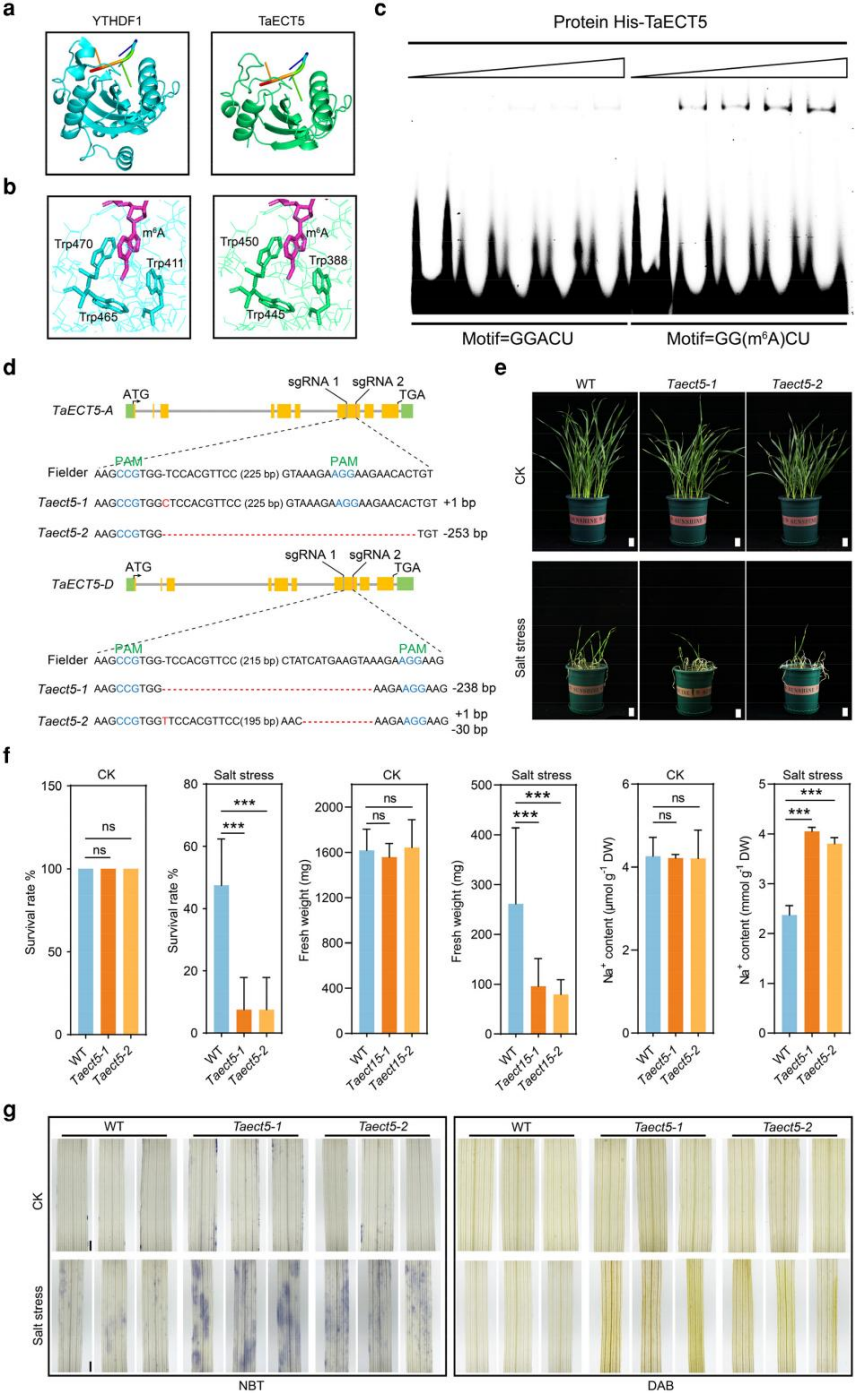
*TaECT5* is involved in salt response in wheat. (a) Structural modeling of HsYTHDF1 and TaECT5 in complex with an m^6^A-modified RNA. (b) Tryptophan residues constituting aromatic cages for m^6^A recognition are labeled. (c) RNA-EMSA was performed to verify the binding of His-TaECT5 to m^6^A-modified RNA. The 5′-FAM-labeled RNA probe (5′-UCUUUGGXCUGACUUGGACUCUUUA-3′) was used at a concentration of 25 nmol, where X represents A or m^6^A. (d) CRISPR/Cas9-mediated target mutagenesis of *TaECT5*. Two CRISPR/Cas9 target sites indicated by sgRNA 1 and sgRNA 2. Exons and other sequences are indicated by boxes and gray lines, respectively. Mutant sequence indicated by red letters and dotted lines. (e) Phenotypic features of WT and *Taect5* under normal and salt stress conditions. (f) Statistics of survival rates, fresh weight, Na^+^ content, in WT, and *Taect5* under normal and salt stress conditions, respectively. (g) NBT and DAB staining in WT and *Taect5* under normal and salt stress conditions, respectively. Scale bar, 1 cm in (e); 200 μm in (g). Data for survival rates are means ± SD with n = 4 and n = 7, respectively, and 5 individual plants per replicate. Data for fresh weight are means ± SD with n = 20 and n = 35, respectively. Data for Na^+^ content are means ± SD with n = 3 and 10 individual plants per replicate. Asterisks indicate significant differences by Student's *t*-test (* *P* < 0.05, ** *P* < 0.01, *** *P* < 0.001).

Following the identification of salt stress-induced dynamics in m^6^A methylation, we sought to identify the regulatory factors driving these changes. The m^6^A epitranscriptomic network is orchestrated by writers, erasers, and readers (Yue et al. 2019; Shen et al. 2023; Song et al. 2024). We analyzed the expression of 89 known m^6^A regulatory genes in wheat roots under salt stress (Yue et al. 2019). Reverse transcription quantitative polymerase chain reaction validation confirmed that 5 were significantly upregulated and 2 were downregulated (Figure S5). A review of their tissue expression profiles revealed that only *TaECT5* (TraesCS7A02G276800), a wheat homolog of *Arabidopsis ECT5*, showed substantial and specific expression enrichment in root tissues (Figure S6a). Sequence alignment of YTH domains showed that TaECT5 shares high sequence homology with mammalian YTHDF1-3 and *Arabidopsis thaliana* ECT2 and ECT8 (Figure S6b). Using the crystal structure of m^6^A-bound YTHDF1 (PDB ID: 4RCJ) as a template, we performed three-dimensional structural modeling via the SWISS-MODEL server. Structural comparison demonstrated that the aromatic cage responsible for m^6^A recognition, which is composed of 3 conserved tryptophan residues, is highly conserved between YTHDF1 (Trp411, Trp465, Trp470) and TaECT5 (Trp388, Trp445, Trp450) (Fig. 6a and b). To further verify this conservation, we carried out RNA electrophoretic mobility shift assay (RNA-EMSA) using recombinant His-tagged TaECT5 purified from *Escherichia coli*. The RNA probes adopted in this assay contained the canonical RRACH motif, a consensus sequence recognized by ECT2 and ECT8 (Cai et al. 2024a, 2024b; Seigneurin-Berny et al. 2024). Electrophoretic mobility shift assay results confirmed that TaECT5 can specifically bind to RNA probes carrying m^6^A-modified RRACH motifs (Fig. 6c).

In hexaploid wheat, *TaECT5* has 3 homoeologous copies: *TaECT5*-*A*, *TaECT5*-*B*, and *TaECT5*-*D* (TraesCS7A02G276800, TraesCS7B02G174500, TraesCS7D02G276800). Notably, only the A and D copies were strongly transcribed in roots (Figure S7). To further investigate its function, we generated *TaECT5* knockout lines (*Taect5*-*aadd*) in the Fielder background using CRISPR-Cas9 (Fig. 6d). Homozygous T_2_ generation lines were used for subsequent phenotypic analysis. Under normal conditions, these knockout lines showed no visible phenotypic differences from the wild type (WT) at the seedling stage. However, under salt stress, the survival rate of *Taect5* seedlings was significantly reduced by 82.4%, and shoot fresh weights decreased by 51.9% and 60.6% compared to WT (Fig. 6e and f). Nitroblue tetrazolium and DAB staining assay showed that a strong signal was detected in *Taect5* (Fig. 6g). Consistently, *TaECT5* knockout plants accumulated significantly higher levels of Na^+^ in shoots (1.7-fold and 1.6-fold of WT). (Figure 6f). These results demonstrate that TaECT5 is required for salt stress tolerance in wheat. To elucidate how TaECT5 regulates salt adaptation, we analyzed the expression of key salt-responsive genes in WT and *Taect5* mutants under stress. In the mutants, 12 salt-responsive genes were significantly upregulated (including the notable transporter *TaHKT1;5-D*), and 4 (including *TaSOS1*) were downregulated (Figure S8). Interestingly, 5 of them harbor m^6^A modification sites (Figure S9a). Further RNA degradation assays showed that TaECT5 binding significantly affects the degradation rate of 3 m^6^A-modified salt-responsive transcripts. Specifically, the absence of functional TaECT5 altered the stability of core salt tolerance genes, leading to disrupted expression patterns of salt-responsive genes in *Taect5* mutant (Figure S9b–d). Collectively, these findings indicate that TaECT5 may modulate the wheat salt stress response by regulating the expression of a suite of core salt-responsive genes.

## Discussion

Gene expression is precisely modulated through a cascade of regulatory events spanning transcriptional initiation to protein synthesis, with epitranscriptomic regulation constituting a central layer of control (Sharma et al. 2023; Shen et al. 2023). This multitiered network enables plants to rapidly adjust their functional proteome in response to environmental stresses such as salinity. In recent years, dynamic RNA modifications, most notably m^6^A, together with their consequent effects on protein-level output, have been established as crucial mechanisms for fine-tuning gene expression during stress adaptation (Gao et al. 2022; Wang et al. 2025a, 2025b, 2025c, 2025d). However, how epitranscriptomic dynamics are functionally linked to transcriptome reprogramming and, ultimately, proteome remodeling during the early salt stress response remains unclear in wheat. To address this, we performed an integrated multi-omics analysis of wheat seedlings roots at 0.5 hpt, combining DRS-based epitranscriptomic and transcriptional profiling, PAL measurement, and quantitative proteomics. Our data revealed that a subset of genes with altered m^6^A levels exhibited no significant changes in mRNA abundance, and these m^6^A-modified genes were highly enriched in core signaling pathways governing the early salt stress response. This evidence demonstrates that the m^6^A dynamics observed at 0.5 hpt represent a direct response to salt stress, rather than a passive concomitant outcome of transcriptional reprogramming. In addition, distinct physiological phenotypes associated with stress responses were already detectable in wheat plants at 0.5 h after salt treatment, further verifying the scientific rationale and rationality of selecting this early time point. This study thereby establishes a comprehensive, multilayered profile linking epitranscriptomic dynamics to transcriptomic and proteomic changes during the early salt stress response in wheat.

m^6^A is a key RNA modification involved in crop growth and stress responses (Zhou et al. 2022). Our global m^6^A mapping via DRS in wheat roots revealed 2 defining features: over 95% of m^6^A peaks localized to the 3′ UTR, and the consensus motif RRACH was conserved between control and salt-stressed samples (Fig. 3a and b, Figure S2b). This pronounced 3′ UTR bias aligns with m^6^A profiles characterized in *Arabidopsis*, rice, maize, and wheat (Luo et al. 2014; Luo et al. 2020; Huang et al. 2022; Qian et al. 2025), underscoring its evolutionary conservation among plants. The enrichment of m^6^A in 3′ UTRs, a region critical for regulating mRNA stability and translational efficiency, suggests its targeted role in posttranscriptional control. Notably, salt stress induced a reduction in the global m^6^A modification ratio (Fig. 3c), a trend consistent with observations in rice seedlings (Qian et al. 2025). This genome-wide attenuation, particularly within the regulatory hotspot of the 3′ UTR, could facilitate rapid transcriptome reprogramming by broadly adjusting the posttranscriptional fate of mRNAs, representing a potential conserved strategy for early salt stress response. Integrated analysis further indicated that changes in m^6^A modification correlated with altered transcriptional abundance of key salt-responsive genes, including *TaHKT1;5*-*D*, *TaPLATZ2*, and *TaGBF1*, thereby supporting a functional link between m^6^A dynamics and the salt stress response.

**Figure 7:**
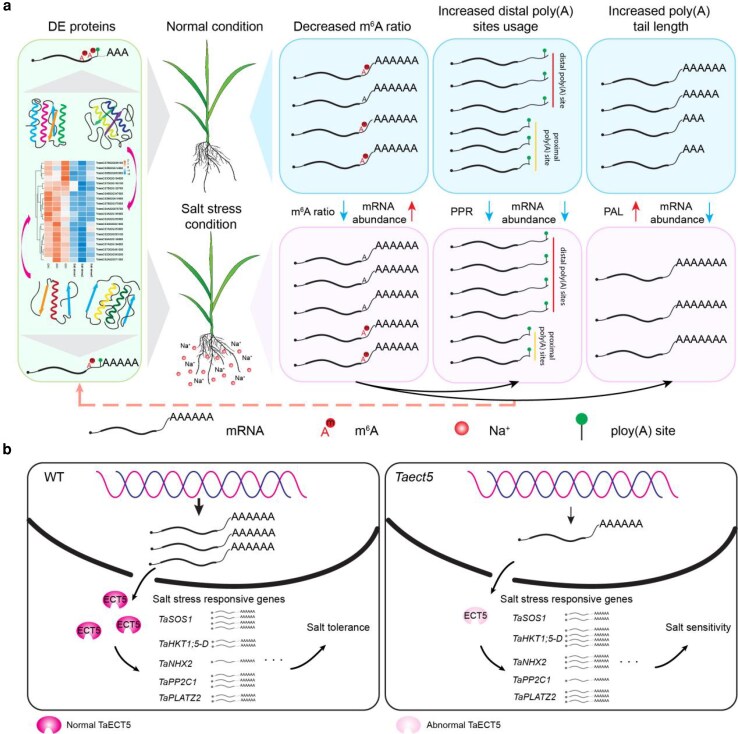
Proposed working models. (a) A model depicting posttranscriptional and protein-level regulatory changes in wheat seedlings during early salt stress response. Salt stress reduces global m^6^A modification levels, thereby positively modulating transcript abundance; promotes distal poly(A) site usage; and elevates poly(A) tail length, which in turn exerts a negative regulatory effect on transcript abundance. Notably, m^6^A-modified mRNAs tend to harbor longer poly(A) tails, and APA may functionally interact with m^6^A modification to fine-tune transcriptome homeostasis. Ultimately, these posttranscriptional regulatory events coordinately modulate protein abundance under salt stress conditions. (b) Under salt stress conditions, loss-of-function mutation of *TaECT5* modulates the mRNA abundance of salt stress-responsive genes, resulting in increased salt sensitivity in wheat.

Alternative polyadenylation is a well-documented mediator of stress-responsive transcriptome dynamics in plants (Téllez-Robledo et al. 2019; Gao et al. 2022; Ma et al. 2022; Ge et al. 2025; Wang et al. 2025a, 2025b, 2025c, 2025d). Our direct quantification revealed a global shift toward distal poly(A) site usage under salt stress (Fig. 4a). Genes exhibiting these differential APA events were functionally enriched in transmembrane transport and oxidative stress responses (Fig. 4b). The preferential selection of distal sites often extends the 3′ UTR, which can introduce or alter cis-regulatory elements affecting mRNA stability and translation. These findings underscore the role of APA in diversifying the salt-responsive transcriptome, thereby contributing to regulatory plasticity under stress. Concurrently, we observed a salt-induced increase in median PAL, corroborating recent reports (Gao et al. 2022). Genes with longer PAL were enriched in salt stress responses, suggesting that PAL extension may stabilize stress-responsive mRNAs to ensure sustained translation. This notion is supported by pronounced PAL lengthening and increased mRNA abundance of known salt-responsive genes like *TaTIFY3B* and *TaSP* (Ma et al. 2015; Liu et al. 2024a, 2024b, 2024c). Furthermore, the correlation between PAL and gene expression aligns with conserved eukaryotic regulation, while the longer PAL of m^6^A-modified genes, which is accentuated under stress, points to potential epitranscriptomic coordination between m^6^A methylation and poly(A) tail dynamics (Fig. 7). This observed convergence suggests that the m^6^A-PAL cross talk may function as an integrated layer of posttranscriptional control, potentially fine-tuning the stability, localization, or translational efficiency of key stress-responsive mRNAs under adverse conditions.

The functional roles of m^6^A readers in plant stress responses represent an emerging area of epitranscriptomic research. Recent studies highlight their regulatory significance, as demonstrated in *Arabidopsis* where readers such as ECT8 and ECT12 modulate mRNA stability during abiotic stress (Amara et al. 2024; Cai et al. 2024a, 2024b; Nguyen et al. 2025). To investigate whether such functional conservation extends to crops, we identified TaECT5, a wheat homolog of the *Arabidopsis* m^6^A reader ECT5, as a salt-induced regulator in roots. Functional analysis demonstrated that *Taect5* knockout mutants exhibit marked hypersensitivity to salt stress, reflected in reduced survival rates, elevated shoot Na^+^ accumulation, and increased ROS level. Furthermore, expression profiling revealed that TaECT5 shapes the salt stress response network by modulating the transcript levels of multiple key regulators. Thus, our study establishes TaECT5 as a critical salt stress tolerance factor in wheat and provides the genetic basis for characterizing its potential role, including its putative function as an m^6^A reader.

Based on the functional divergence among ECT family readers, we propose a context-dependent model for TaECT5. It may promote the turnover of target transcripts for genes it represses while potentially stabilizing mRNAs for genes it upregulates. This functional duality is consistent with the broader YTHDF protein family. The functional redundancy of ECT5 with ECT2, a factor known to accelerate the decay of m^6^A-modified viral RNAs in *Arabidopsis* (Martínez-Pérez et al. 2023), suggests that mRNA degradation may represent one plausible mechanism through which TaECT5 regulates salt tolerance. Prime candidate targets include critical regulators like *TaSOS1*, whose altered expression in *Taect5* mutants, and RNA degradation assays supports this hypothesis (Figures S8 and S9). However, definitive validation would require identifying the direct RNA targets of TaECT5 and confirming the functional consequence of its binding. Resolving these questions will be essential to substantiate the proposed model and to define the precise mechanistic role of TaECT5 within the epitranscriptomic regulatory network. In summary, this study identifies TaECT5, a putative m^6^A reader and homolog of *Arabidopsis* ECT5, and defines an epitranscriptomic framework for early salt stress adaptation in wheat.

## Materials and methods

### Salt treatment and sample collection

Wheat seeds were harvested from experimental fields located in Weifang, Shandong province, China. Following germination, uniform seedlings were transferred to the Hoagland nutrient solution (pH 5.8) and cultured in a light incubator under controlled conditions: 28℃ with a 16-h light/8-h dark. For salt stress treatment, 2-week-old seedlings were exposed to 100 mM NaCl solution for 0.5 h. Wheat roots were sampled prior to treatment as the control group (0 h, CK). All collected samples were immediately snap-frozen in liquid nitrogen and stored at −80℃ until subsequent DRS and 4D data-independent acquisition (4D-DIA) proteomics analyses.

### Nanopore DRS analysis

Direct RNA sequencing was performed by Annoroad Gene Technology (Beijing, China) Co., Ltd. In brief, total RNA from CK and salt-stressed wheat roots was extracted using the Plant RNA Kit (Omega, R6827), and Poly(A) RNA was enriched with the NEBNext® Poly(A) mRNA Magnetic Isolation Module (NEB, E7490). Libraries were prepared using the SQK-NBD114.24 kit. 500 ng of poly(A) RNA was prepared and ligated to the RT adapter. Then, first-strand cDNA was synthesized and the resulting products were purified with AM Pure beads. Then, all libraries loaded onto FLO-PRO114M flow cells for sequencing on the PromethION platform (3 biological replicates). Raw fast5 data were converted to fastq format via base calling with Dorado (version 0.7.3) (https://github.com/nanoporetech/dorado).

### Differential expression gene analysis from DRS

Filtered sequences were aligned to the reference genome using minimap2 (version 2.17-r941) (Qian et al. 2025) with parameters set as -ax splice -uf -k14. The aligned BAM files were then converted into BED12 format the samtools (Li et al. 2009). Gene and chromosomal annotation information was added to the BED12 files using the identify_gene_isoform.py script, followed by clustering and redundancy removal with the collapse module to obtain nonredundant consensus isoforms. DESeq2 (version 1.26.0) software was used to identify DEGs (|log_2_ (fold change)| ≥ 2 and *P_adj_* < 0.05) in salt stress versus CK comparison.

### m^6^A^6^a modification analysis

Dorado (model: rna004_130bps_sup@v5.0.0) (https://github.com/nanoporetech/dorado) was used for identifying m^6^A modification site. Modified-base detection was performed using Dorado’s built-in modified-base models with default parameters. The modification ratio was calculated by the following formula: mod-reads/(un-mod-reads + mod-reads). Fisher's extract test was used to identify differential methylation sites, with significance criteria set at difference in m^6^A ratio ≥ 0.05 and *P* value < 0.05.

### Poly(A) site analysis

After aligning sequencing reads to the reference genome with minimap2, the aligned end positions were extracted from the resulting BAM files. Poly(A) sites were then identified using the QuantifyPoly(A) tool (Ye et al. 2021), with PAC (poly(A) cluster) expression quantified as the number of poly(A) Tag reads within a 24-nucleotide window. For genes containing multiple PACs, the 2 most abundant PACs were selected as representative proximal and distal poly(A) sites. The proximal site was defined as the upstream site relative to the transcription direction (closer to the 5′ end of the transcript), whereas the distal site was defined as the downstream site (closer to the 3′ end of the transcript). The proximal PPR was calculated as PPR = proximal PAC reads/(proximal PAC reads + distal PAC reads). Differences in poly(A) site usage between treatment and control groups were evaluated using Fisher's exact test based on proximal and distal PAC read counts. Genes showing an absolute change in proximal PPR exceeding 10% and *P* value < 0.05 were considered differential APA events.

### Identification of differential PAL

Poly(A) tail lengths were estimated directly from raw nanopore signals during basecalling using Dorado (model: rna004_130bps_sup@v5.0.0) with the –estimate-poly-a option. The Mann–Whitney *U* test was used to analyze differences in PAL between the CK and salt stress groups. Poly(A) tail lengths with FDR < 0.05 and |log_2_ (fold change)| ≥ 0.585 were considered as significantly different.

### Proteomic analysis

Total proteins were extracted from samples using SDT lysis buffer (4% SDS, 100 mM Tris-HCl, pH 7.6). All samples were digested with trypsin via the filter-aided proteome preparation (FASP) method. Digested peptides were desalted using C18 cartridges, lyophilized, and reconstituted in 40 μL 0.1% formic acid (Wisniewski et al. 2009). After adding iRT standard peptides, samples were analyzed by DIA mass spectrometry on an Astral high-resolution mass spectrometer coupled with a Vanquish Neo nano-Liquid Chromatogram system. Raw data were processed using DIA-NN software. A differential expressed protein was defined with |log_2_ (fold change)| ≥ 0.585 and *P* value < 0.05.

### Gene Ontology enrichment

Gene Ontology enrichment analysis was performed using the clusterProfiler package (v4.12.6) (Wu et al. 2021) in R (version 4.4.1), with gene annotations obtained from the IWGSC v1.1.

### Enzyme activity detection

Peroxidase activity was detected following the instructions of the POD Activity Determination Kit (Solarbio, China). Briefly, 0.1 g of tissue was ground into a fine powder in liquid nitrogen, and the resulting homogenate was extracted and processed according to the kit instructions. Peroxidase activity was measured spectrophotometrically at 470 nm. Similarly, SOD activity was assayed using the SOD Activity Determination Kit (Solarbio, China). A 0.1 g of tissue samples were prepared as per the kit protocol, and SOD activity was determined at 450 nm.

### Na^+^ content determination

For Na^+^ content determination, salt-treated and non-treated shoot tissues were dried to constant weight, ground into powder, and 0.1 g samples were digested in 10 mL HNO_3_ solution using a microwave digester. Diluted to 10 mL with ddH_2_O, the samples were analyzed for Na^+^ content by inductively coupled plasma mass spectrometry (ICP-MS) with 3 biological replicates.

### Nitroblue tetrazolium chloride (NBT) and 3,3′-diaminobenzidine (DAB) staining assay

Root and shoot tissues were harvested and longitudinally sectioned into halves using a sharp scalpel. Subsequently, the prepared samples were immersed separately in reaction buffers containing 0.5 mg•mL^−1^ NBT and 1 mg•mL^−1^ DAB, respectively, and incubated at 37℃ in the dark for the histochemical detection of superoxide anions and hydrogen peroxide. After staining, the samples were fixed in 70% (v/v) ethanol at room temperature.

### Gene expression analysis by RT-qPCR

For RNA extraction, frozen tissues were ground to a fine powder in liquid nitrogen, and total RNA was purified using the FastPure Universal Plant Total RNA Isolation Kit (Vazyme, China). RNA concentration and purity were determined using a NanoDrop 2000 spectrophotometer (Thermo Fisher Scientific, USA). First-strand cDNA was reverse-transcribed from 2 μg of DNase I digested total RNA using the All-In-One 5X RT MasterMix (abm, Canada) according to the instructions. Reverse transcription quantitative polymerase chain reaction was performed following the method described by Zhao et al. (2024). Each assay included 3 biological replicates, with *TaActin* serving as the internal reference gene for normalization. Primer sequences are provided in Table S1.

### Vector construction and plant transformation

To generate genome-edited wheat lines, single-guide RNAs (sgRNAs) targeting the homologous regions of *TaECT5* were designed using the CRISPRdirect tool (http://crispr.dbcls.jp/). Two sgRNAs were selected: sgRNA1 (sequence: 5′-TGGTCCACGTTCCACCAGGC-3′) and sgRNA2 (sequence: 5′-GGCCTATCATGAAGTAAAGA-3′). The sgRNA-expression cassettes were cloned into pBUE413 vector, which were subsequently introduced into *Agrobacterium tumefaciens* strain EHA105. Transgenic wheat plants were generated through *Agrobacterium*-mediated transformation using the cultivar Fielder as the genetic background. T_0_ transgenic plants harboring loss-of-function mutations in *TaECT5* were identified by Sanger sequencing and self-pollinated to produce T_1_ progeny. T_1_ plants were further self-pollinated to obtain homozygous double mutants of *TaECT5*.

### RNA-EMSA

To explore the binding capability of TaECT5 toward target RNA, RNA-EMSA was conducted with the RNA-EMSA Kit (Thermo Scientific, 20158) following standard manufacturer protocols. For EMSA reactions, each system contained 1 μL of FAM-labeled RNA probe (final concentration of 25 nM), 2 μL recombinant protein at varying concentrations, 2 μL 10× binding buffer, 2 μL 50% glycerol, and 13 μL nuclease-free water. Samples mixed with RNA loading buffer were separated on 6% TBE gels. Electrophoresis was performed at 90 V for 90 min in pre-cooled 0.5× TBE buffer (5 g/L Tris, 2.75 g/L boric acid, 50 mM EDTA, pH 8.0) under ice bath conditions. Images were finally captured using a ChemiDoc imaging system (Bio-Rad).

### mRNA stability assay

To examine mRNA stability, we applied 200 μM actinomycin D to suppress cellular transcription. Seedlings of WT and *Taect5* grown in Hoagland nutrient solution were subjected to actinomycin D treatment. Following a 1-h incubation, samples were harvested as the control, and further collections were conducted at 2, 4, and 6 h. Three biological replicates were included for all time points. Reverse transcription quantitative polymerase chain reaction was used to detect the abundance of target transcripts, and the ACTIN gene was used as the reference for normalization.

### Accession numbers

Sequence data from this article can be found in EnsemblPlants (http://plants.ensembl.org/index.html) data libraries under accession numbers: *TaECT5-A* (TraesCS7A02G276800), *TaECT5-B* (TraesCS7B02G174500), and *TaECT5-D)* (TraesCS7D02G276800).

## Supplementary Material

kiag540_Supplementary_Data

## Data Availability

All data supporting the findings of this study are available within the paper and within its supplementary materials published online. Raw sequencing data and proteomic data have been deposited in the Genome Sequence Archive (GSA, https://www.cncb.ac.cn/) and the PRIDE database (https://www.iprox.cn/), with accession numbers CRA037282 and PXD081584, respectively.
