# Supplementary material for: Dissecting wheat epitranscriptome and proteome under salt stress characterizes an m6A reader gene vital for salinity adaptation
Source: Plant Physiol. 2026 Jul 24;201(4):kiag540. doi: 10.1093/plphys/kiag540 (PMC13440310; doi:10.1093/plphys/kiag540)
Supplement: Supplementary file 1 [file kiag540_supplementary_data.zip › Supplemental Figures.pdf]

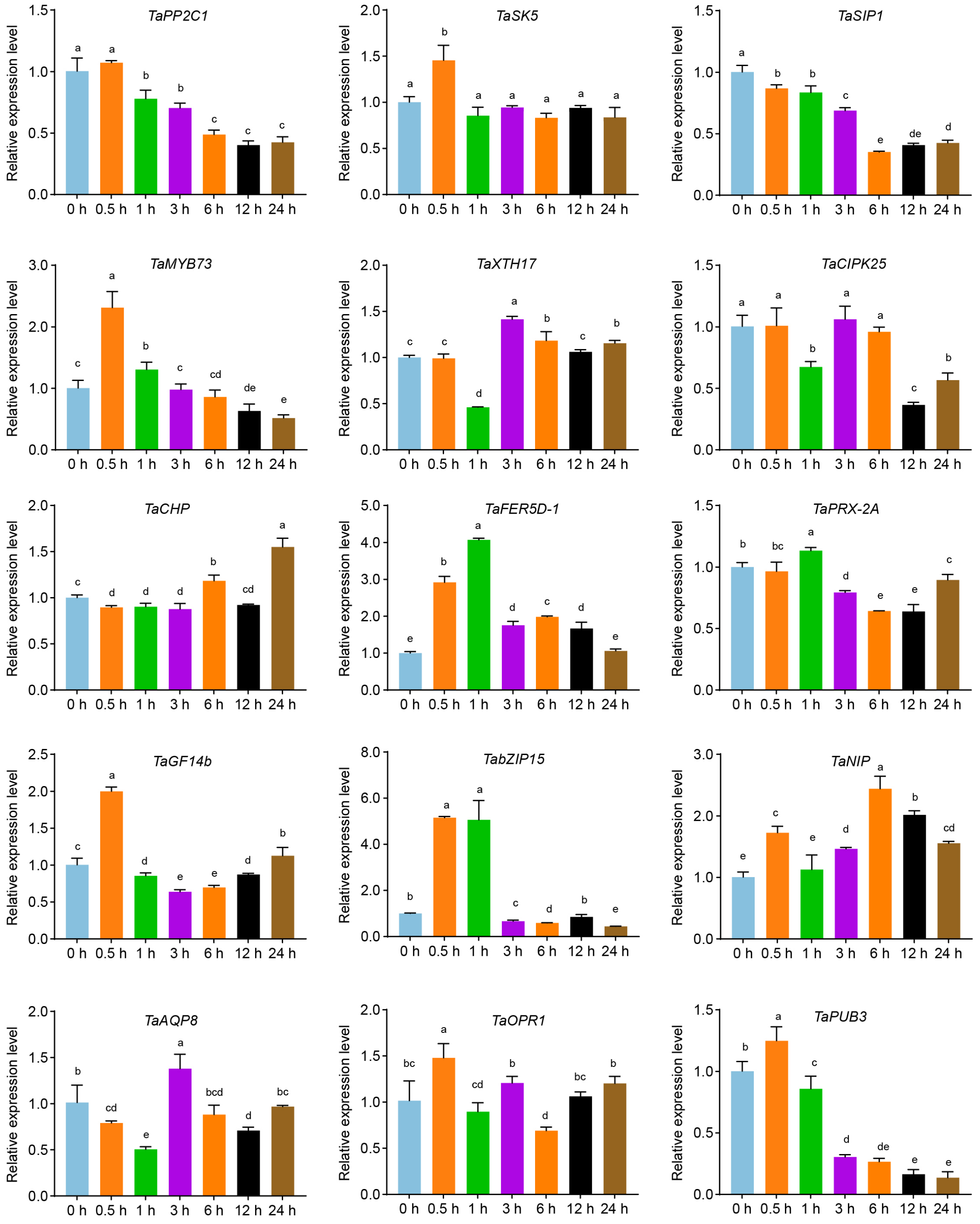

Supplemental Figure S1. RT-qPCR analysis of fifteen well-characterized salt stress response genes.Note: Values are shown as mean ± SD with three independent biological replicates. Statistical significance of differences between treatments was determined using one-way analysis of variance (ANOVA). Columns labeled with the same lowercase letter are not significantly different at $P < 0.05$, different lowercase letters indicate statistically significant differences ($P < 0.05$).

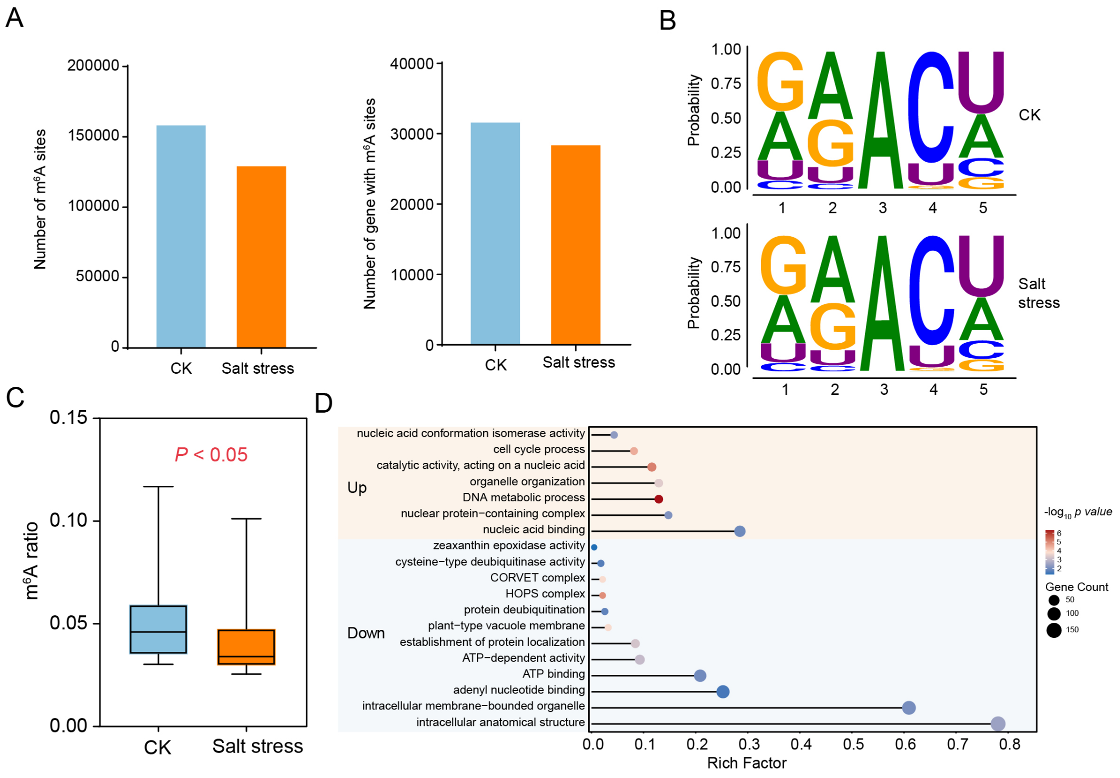

Supplemental Figure S2. Dynamics changes of m^{6}A modification under salt stress. (A) Statistics of m^{6}A sites and genes with m^{6}A sites under normal and salt stress conditions. (B) Sequence logo representing consensus motifs found in CK and salt stress samples. (C) m^{6}A ratio of representative salt stress response genes under normal and salt stress conditions. Boxes denote the interquartile range, where the bottom and top boundaries correspond to the 25th and 75th percentiles, respectively. The horizontal line within each box represents the median value, and whiskers extend to the minimum and maximum values of the dataset. Statistical significance was assessed using the Student's *t*-test. (D) GO enrichement analysis of m^{6}A DMSs in response to salt stress.

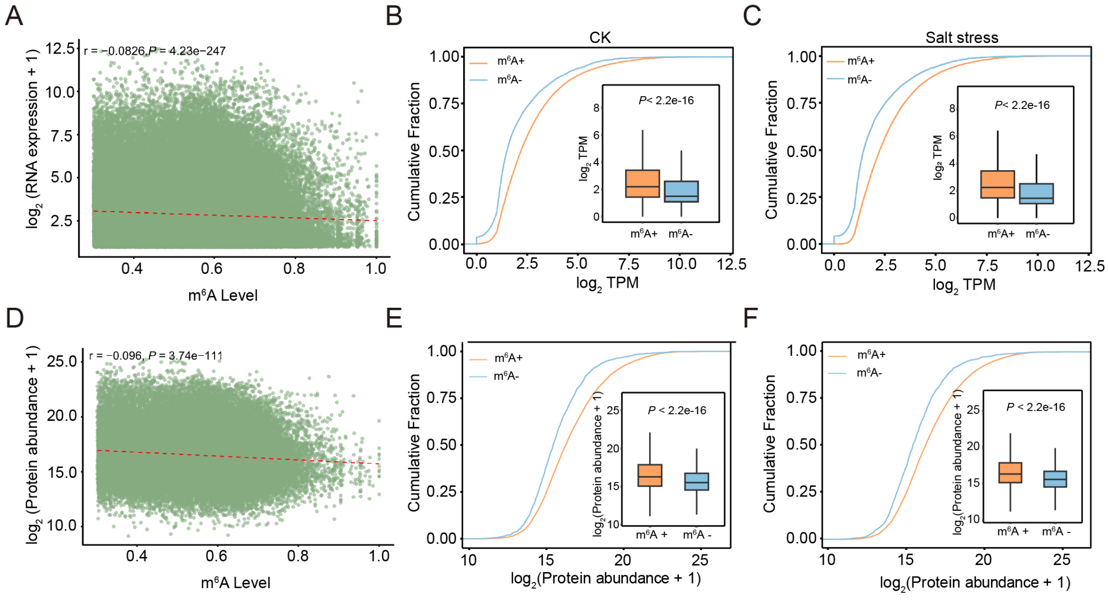

Supplemental Figure S3. Correlation of m^6A modification and gene expression and protein abundance. (A) Scatter plot shows associations of m^6A modification and gene expression. (B-C) Cumulative combined boxplot show distribution of changes in transcript expression between m^6A-modified (m^6A+) and non-m^6A-modified (m^6A-) genes under normal and salt stress conditions, respcetively. (D) Scatter plot shows associations of m^6A modification and protein abundance. (E-F) Cumulative combined boxplot show distribution of changes in protein abundance between m^6A+ and m^6A- protein under normal and salt stress conditions, respcetively. In (B-C) and (E-F), boxes denote the interquartile range, where the bottom and top boundaries correspond to the 25th and 75th percentiles, respectively. The horizontal line within each box represents the median value, and whiskers extend to the minimum and maximum values of the dataset. Statistical significance was assessed using the Student's *t*-test.

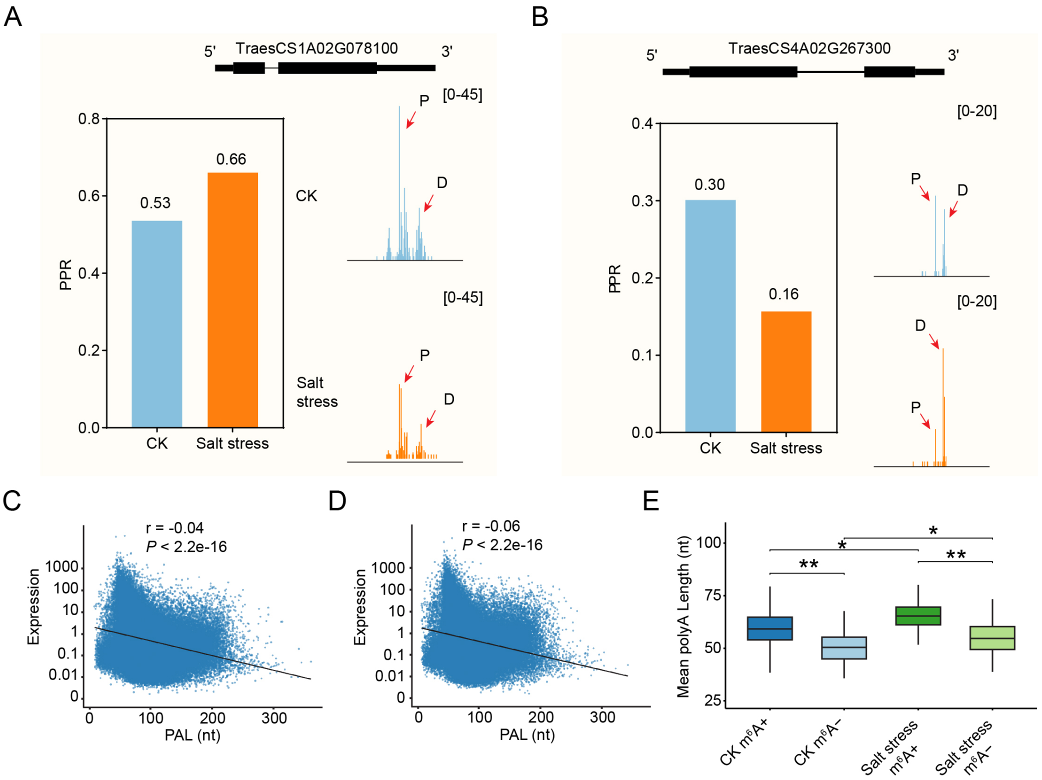

Supplemental Figure S4. Poly(A) site usage and PAL dynamic changes in response salt stress. (A) Wiggle plot and histogram shows increased PPR in *TaPRX52*. (B) Wiggle plot and histogram shows increased PPR in *TaRLK1*. (C-D) Scatter plot shows associations of PAL and gene expression under normal and salt stress conditions, respectively. (E) Average PAL of m^{6}A modified transcripts in CK and salt stress. Boxes denote the interquartile range, where the bottom and top boundaries correspond to the 25th and 75th percentiles, respectively. The horizontal line within each box represents the median value, and whiskers extend to the minimum and maximum values of the dataset. Statistical significance was assessed using the Student's *t*-test. * $P < 0.05$, ** $P < 0.01$. Letter D represents distal, letter P represents proximal.

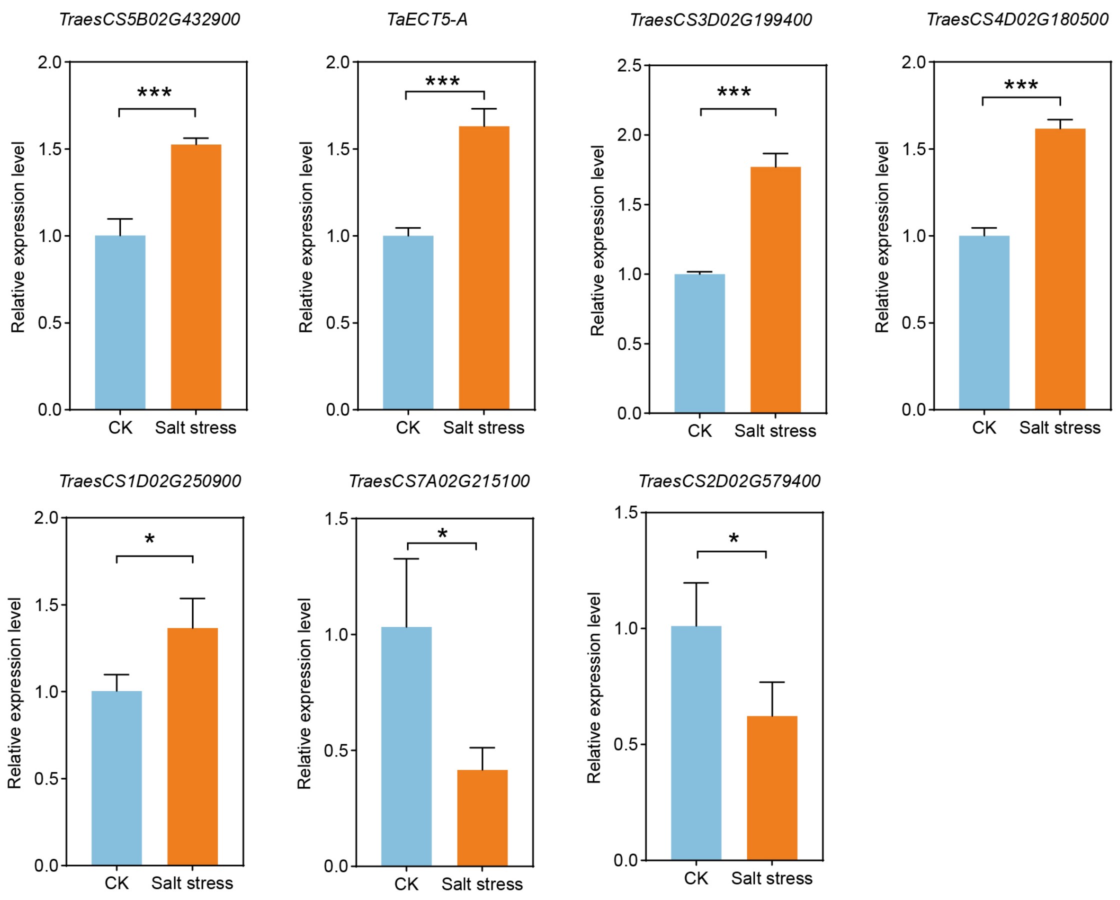

Supplemental Figure S5. RT-qPCR analysis of seven genes involved in m^{6}A modification under normal and salt stress conditions. Values are shown as mean ± SD with three repeats, and asterisks indicate significant differences by Student's t-test (* *P* < 0.05, *** *P* < 0.001).

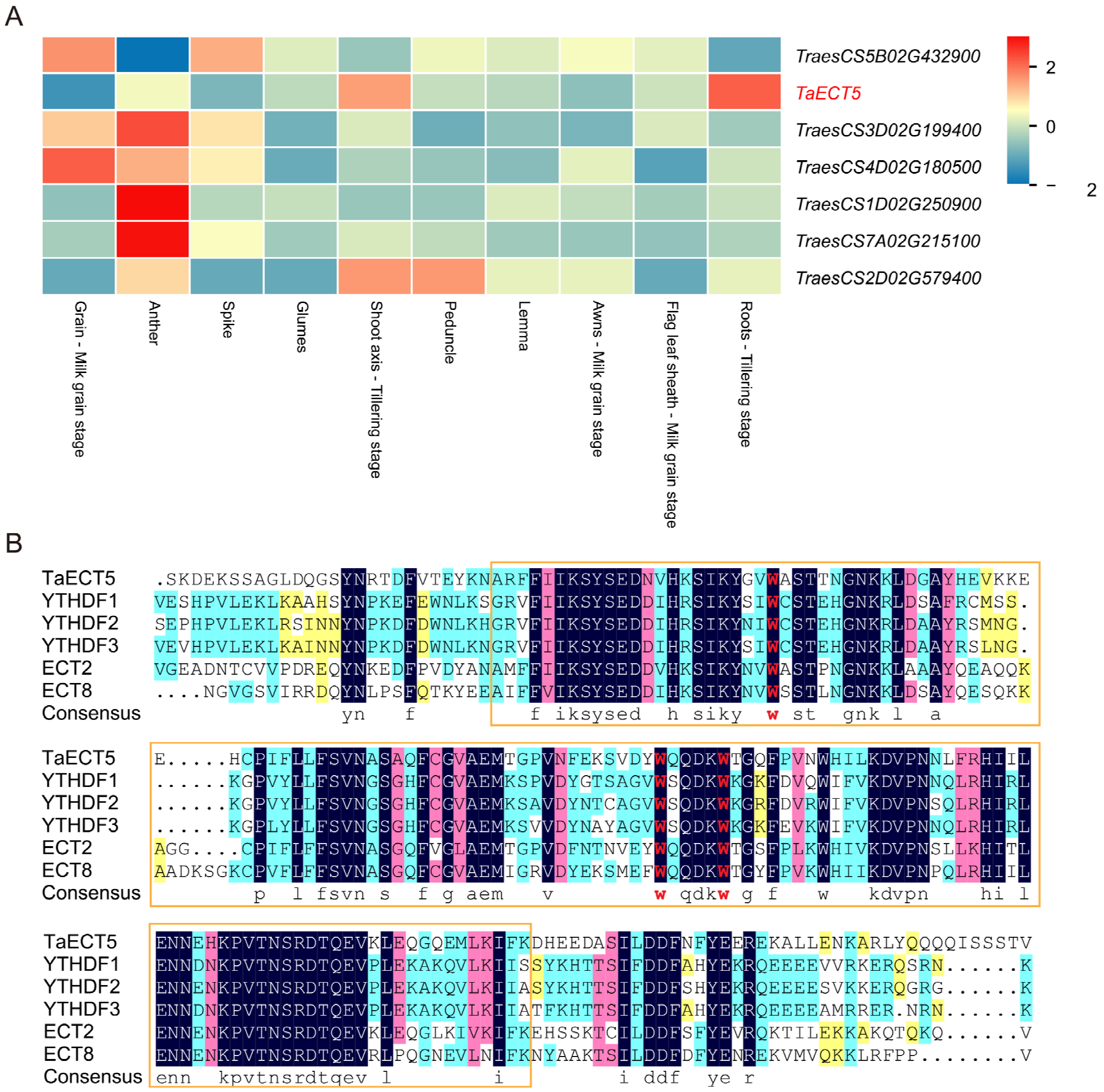

Supplemental Figure S6. Tissue-specific expression of seven genes associated with m^{6}A modification, and YTH domain sequence alignment of TaECT5 with mammalian YTHDF1-3 and Arabidopsis ECT2/ECT8. (A) Expression patterns of seven genes related to m^{6}A modification. (B) Multiple sequence alignment reveals high conservation of the YTH domain. The orange box marks the YTH domain, and conserved tryptophan residues are highlighted in bold red letter W.

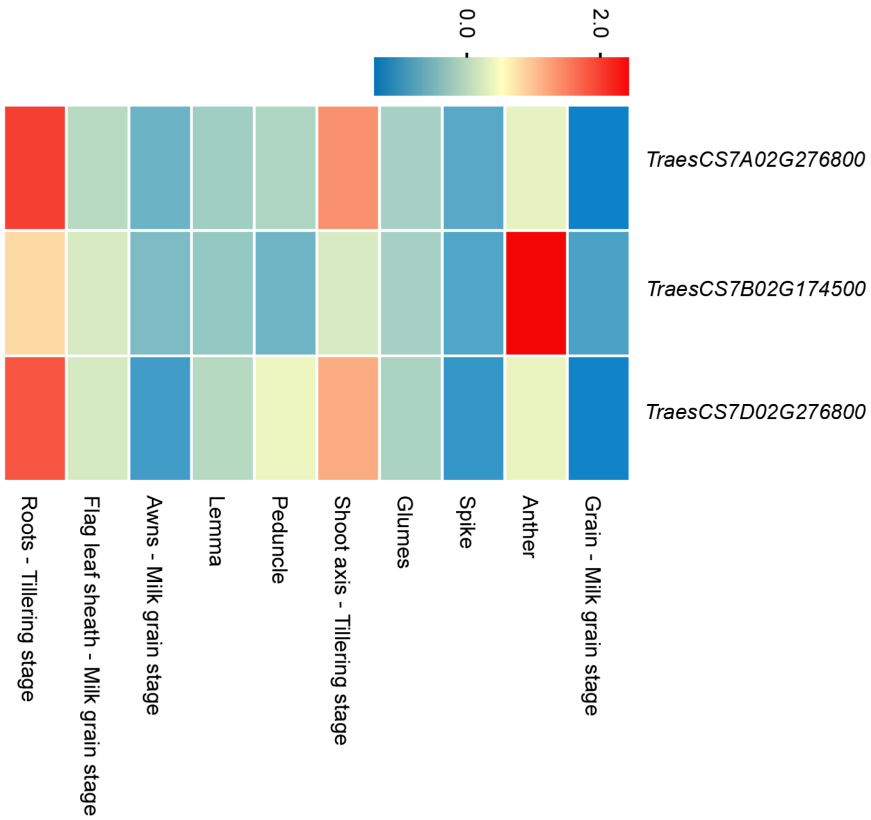

Supplemental Figure S7. Expression pattern of three homoeologous copies of *TaECT5* in different tissues.

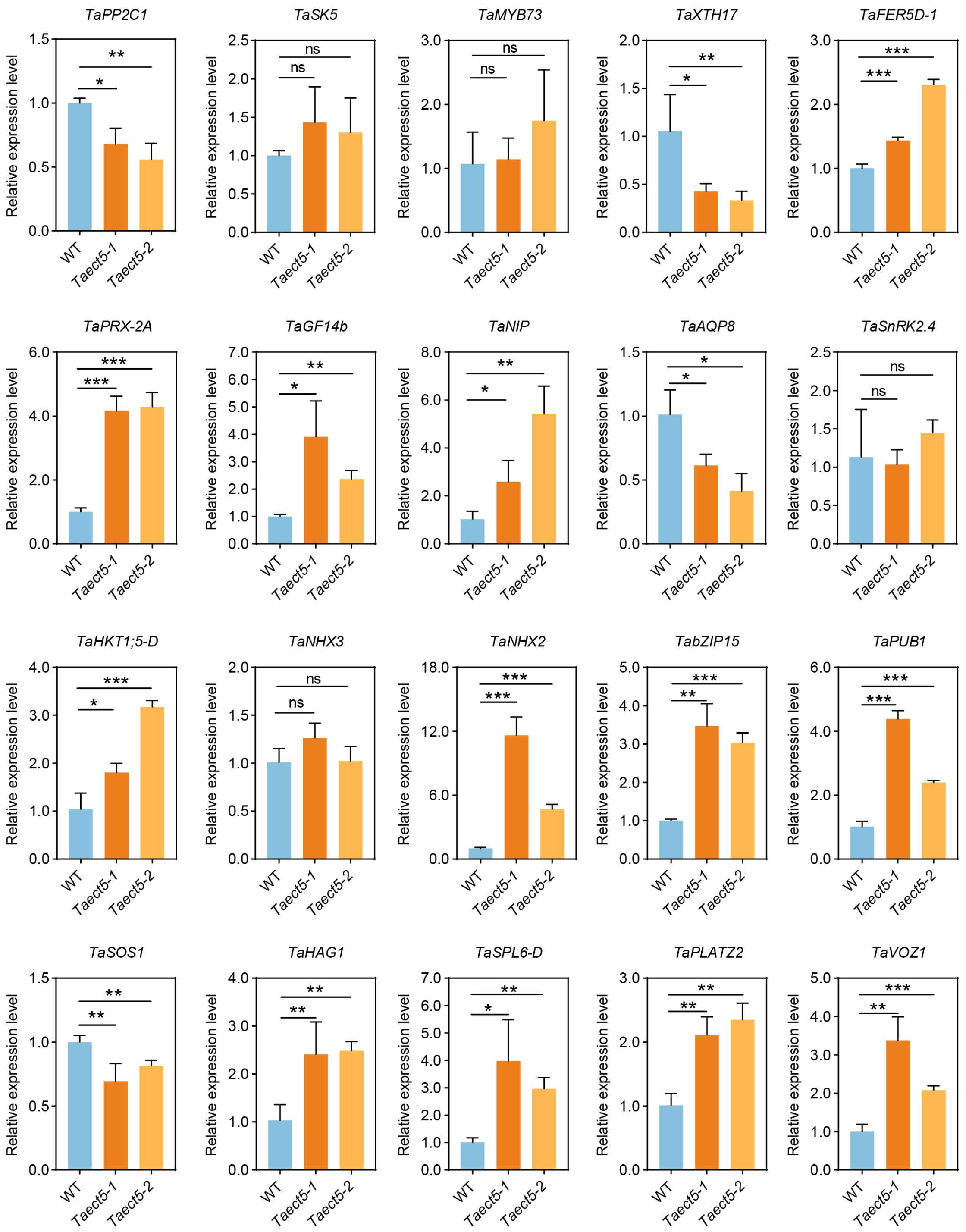

Supplemental Figure S8. RT-qPCR analysis of salt response related genes in WT, *Taect5-1*, and *Taect5-2* under salt stress condition. Values are shown as mean ± SD with three repeats, and asterisks indicate significant differences by Student's t-test (ns, not significant, * $P < 0.05$, ** $P < 0.01$, *** $P < 0.001$).

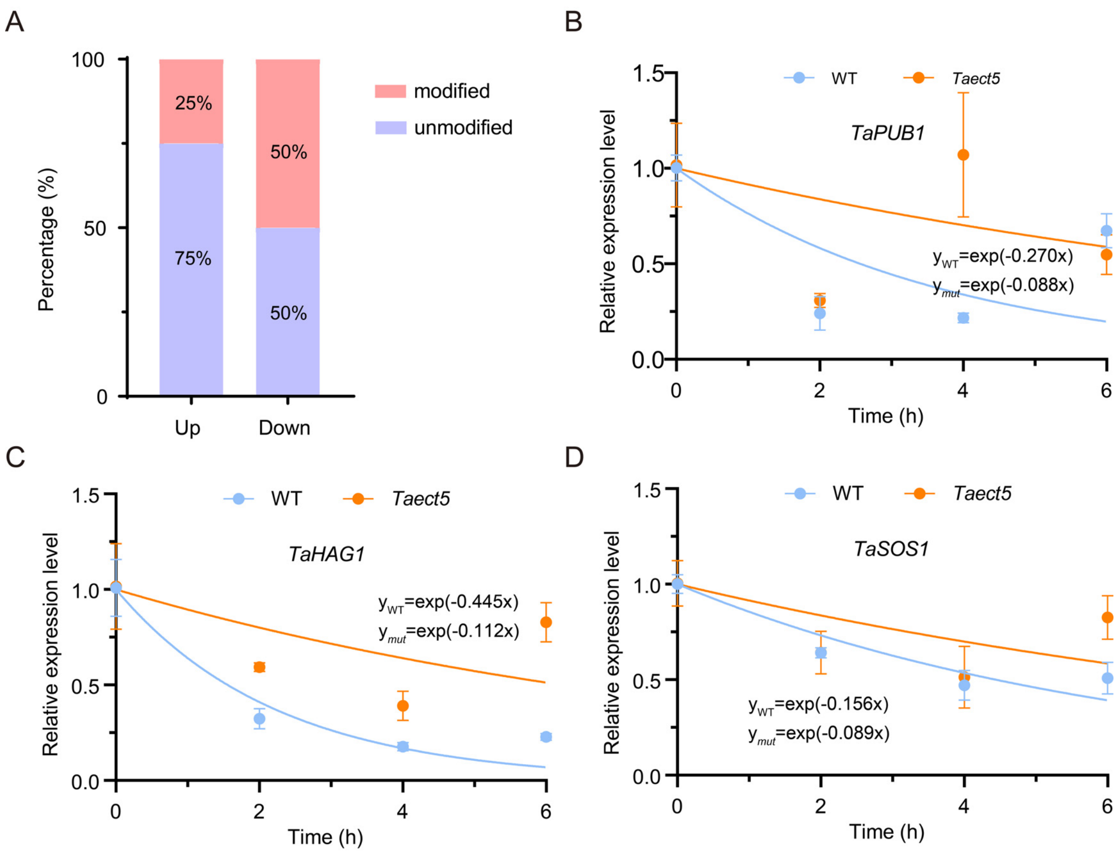

Supplemental Figure S9. The proportions of 16 salt-related DEGs with or without m^{6}A modification, and mRNA half-lives of the indicated genes in WT and *Taect5* seedlings. (A) Proportion of 16 salt-related DEGs with or without m^{6}A modification. (B-D) The mRNA half-lives of *TaPUB1*, *TaHAG1*, and *TaSOS1* transcripts in WT and *Taect5* seedlings. Values are shown as mean ± SD with three repeats.
